# Βotanical Origin Confirmation and Adulteration Testing of Monofloral Honey Using ATR-FTIR Spectroscopy in Combination with Pattern Recognition and Dimension Reduction Techniques

**DOI:** 10.3390/foods15091544

**Published:** 2026-04-29

**Authors:** Dimitrios G. Lazaridis, Vassilios K. Karabagias, Sofia Karabournioti, Aris E. Giannakas, Ioannis K. Karabagias

**Affiliations:** 1 Department of Food Science & Technology, School of Agricultural Sciences, University of Patras, G. Seferi 2, 30100 Agrinio, Greece; up1076474@ac.upatras.gr (D.G.L.); vkarampagias@upatras.gr (V.K.K.); agiannakas@upatras.gr (A.E.G.); 2 Attiki Bee Culturing Co.–Alex. Pittas S.A., 9 Protomagias Street, 14568 Athens, Greece; skar@attiki-pittas.gr

**Keywords:** honey, melissopalynology, ATR-FTIR, authentication, adulteration, chemometrics

## Abstract

The present study aimed to investigate whether Attenuated Total Reflectance-Fourier Transform Infrared (ATR-FTIR) spectroscopy could be effectively applied for the botanical origin confirmation of monofloral (fir, thyme, pine) and flower/polyfloral (flower, citrus, asfaka, and mixtures) honey in accordance with melissopalynological analysis, and to unveil the adulteration of monofloral honey with flower/polyfloral honey. Fifty-nine samples were subjected first to melissopalynological analysis to record the dominant pollen flora. Afterwards, ATR-FTIR analysis identified the dominant spectral regions of interest. Among them, 3300–3200 cm^−1^, 2970–2920 cm^−1^, 1730–1600 cm^−1^, 1420–1410 cm^−1^, 1390–1380 cm^−1^, 1380–1330 cm^−1^, 1260–1225 cm^−1^, 1210–1180 cm^−1^, 1150–1130 cm^−1^, 1100–1010 cm^−1^, and 950–750 cm^−1^ showed a differentiation potential. Pattern recognition [multivariate analysis of variance (MANOVA)/linear discriminant analysis (LDA) and dimension reduction (factor analysis)] techniques resulted in 100% classification of samples by botanical origin, with the most significant factor parameters being the regions of 1730–1600 cm^−1^, 1420–1410 cm^−1^, and 950–750 cm^−1^, which indicate the presence of water, carbohydrates, ketones, amino acids, and organic acids. Fir, thyme, and pine samples were also adulterated with the batch of flower/polyfloral honey samples (20% *w*/*w*), and ATR-FTIR, in combination with the aforementioned multivariate techniques, differentiated the adulterated samples from monofloral samples with an overall cross-validation prediction rate of 91.2% based on LDA. ATR-FTIR, when combined with chemometrics, can be a rapid analytical technique for confirming the botanical origin and adulteration of monofloral honey with polyfloral honey, with an error rate below 9%.

## 1. Introduction

The *Apis mellifera* honeybees collect the nectar of flowers or honeydew from the honeydew secretions of conifer trees, and after the addition of their enzymes, convert them into honey, which is then kept in their beehives to mature [1]. The basic components of honey are mainly sugars (approx. 77% to 78%), especially the monosaccharides fructose and glucose, and moisture (aprox. 17%). Honey also contains organic acids, such as gluconic acid; amino acids, mostly proline; proteins; numerous minerals, including iron, potassium, magnesium, and calcium; vitamins; and other phytochemical compounds, resulting in its antioxidant and antimicrobial activity along with its considerable nutritional value [2].

Honey is botanically classified as monofloral when it is produced from the nectar or honeydew of a single botanical species, or based on the presence of predominant pollen grains, and as polyfloral when it originates from more than one botanical species [3]. The official method for determining the botanical origin of honey is melissopalynology, which examines the specific pollen morphology through microscopic analysis [4]. Apart from its botanical origin, several factors affect honey quality and stability, including the geographical origin and the climate of the wider geographic area, the type of bees (stingless or *Apis mellifera*), as well as processing and manufacturing practices, handling, packaging, and storage conditions [5]. Therefore, in recent years, there has been extensive work by the official authorities and researchers to authenticate honey. We should keep in mind that authentic products command a higher price in the domestic and international markets [6]. Regarding the research community, extensive work has been carried out using alternative methods such as conventional physicochemical parameter analysis combined with sensory examination [5], chromatographic techniques including the determination of volatile compounds [7], phenolic compounds [8], minerals and other bio-metabolites [9], spectrometric and isotopic fingerprints [10,11,12], using supervised and unsupervised statistical techniques [13], to characterize in depth the botanical and geographical origin of nectar or honeydew honey and test for adulteration [14,15], given that melissopalynology has certain drawbacks. For example, it has been reported as a time-consuming methodthat requires exceptional experts for the evaluation and interpretation of results, whereas in certain cases where the pollen is in low percentages (under-represented), it cannot be robustly implemented [16].

Speaking of adulteration of honey, it can occur through direct and indirect practices. The direct practices include dilution with cheap sweeteners (e.g., corn syrup, glucose, and fructose syrups, etc.) or mixed honeys, along with incorrect labeling of the geographical and botanical origins. The indirect practices include artificial feeding of bees in beehives and harvesting honey with a higher moisture content than usual (unripe honey) [17,18].

In the last ten years, multivariate statistical methods that were initially created for analytical chemistry have gained significant traction in the fields of food science and technology. Chemometrics proves to be especially beneficial when managing extensive and intricate datasets that include various types of samples, quantities, and responses. These methodologies aid in verifying geographical origins, agricultural practices, and identifying adulteration in premium food products and can be grouped into supervised (multivariate discriminant analysis, logistic regression, neural networks, regression/classification trees, etc.) and unsupervised statistical techniques (cluster analysis, factor analysis, principal component analysis, etc.) [13,19]. In summary, chemometrics serves as a crucial instrument for tackling complex, multifactorial issues in food science through a comprehensive approach. Regulatory agencies and industries tasked with monitoring food quality, evaluating raw materials, and optimizing processes should adopt chemometrics, especially when dealing with high-dimensional data of foods [13].

Fourier Transform Infrared Spectroscopy (FTIR) is a simple technique that, when combined with pattern recognition, can identify the origin and adulterants of honey. The chemical composition of honey can be used as an identification method, since it yields a unique IR spectrum. FTIR reads characteristic IR absorption bands at a specific frequency range that correlate to stretching of functional groups, thus providing a molecular fingerprint [10]. FTIR instruments are affordable, reagent-free, rapid, and straightforward to operate. The technique needs no sample preparation, safeguards the sample, and yields information on many components per spectrum. Hence, the use of FTIR may provide a benefit over other methods [20]. However, we should keep in mind the spectral interference or sensitivity issues when applying this technique in food analysis [20].

To our knowledge, the literature is scarce on the use of ATR-FTIR to confirm the botanical origin of Greek monofloral honey [7,21]. Furthermore, research is limited on testing for adulteration with flower/polyfloral honey, rather than the frequently studied commercial syrups [22], based primarily on the transmittance values of specific spectral regions. In addition, the article aims to guide further researchers in choosing the most appropriate spectral regions when applying ATR-FTIR in combination with chemometrics, which could probably be used as key spectral fingerprints for the authentication and adulteration control of honey. This constitutes the novelty of the present research on the authentication/adulteration scenario of Greek honey.

## 2. Materials and Methods

### 2.1. Honey Samples

Fifty-nine honey samples were collected during the harvesting period of 2025 from beekeepers cooperating with Attiki Bee Culturing Co.–Alex. Pittas S.A. (Athens, Greece). The samples were collected from different geographical regions in Greece (Table 1) and divided into four groups: flower/polyfloral (16 samples, consisting of citrus, flower, flower/thyme, asfaka, and flower/asfaka), fir (13 samples), thyme (15 samples), and pine (15 samples). All samples were stored in metallic containers protected from light at room temperature (19 ± 1 °C) and analyzed soon after shipment to the laboratory.

### 2.2. Chemicals and Reagents

The study used no chemicals or reagents for the ATR-FTIR analysis, pure distilled water to dissolve honey, supporting green techniques for the analysis of foods.

### 2.3. Melissopalynological Analysis

The botanical origin of the samples was confirmed by melissopalynological analysis, according to von der Ohe et al. [4]. More specifically, 10 g of each honey sample was diluted in 20 mL of distilled water and centrifuged at 3000 rpm for 10 min. The sediment was dried at 40 °C and mounted with Entellan Rapid (Merck, 1.07961.0500) mounting medium for microscopical analysis. The honeydew elements and pollen grains were counted and identified in 20 optical areas at 200× magnification using an OLYMPUS BX 40 light microscope (Tokyo, Japan).

### 2.4. ATR-FTIR Analysis

#### 2.4.1. Preparation and Analysis of Honey Samples

Approximately 20 g of each honey sample was transferred to a 100 mL beaker and heated slightly in a water bath at 37 ± 1 °C with continuous stirring for 2 min. This process was performed to decrystallize the sugars in the honey and ensure they would not affect the subsequent measurements. The ATR-FTIR analysis was carried out using a Shimadzu IRSpirit-TX spectrophotometer (Shimadzu, Kyoto, Japan) following the method described previously [23]. One drop of each honey sample was placed on the ATR receptor, and after 64 scans at a resolution of 4 cm^−1^, the spectra were recorded over the wavelength range 4000–400 cm^−1^. The instrument was calibrated with a baseline correction after every 10 measurements. Each sample was analyzed in duplicate.

#### 2.4.2. Preparation and Analysis of Adulterated Honey Samples

The adulteration of pine, fir, and thyme honey with flower honey was carried out as follows. Firstly, a batch sample from all 16 flower honeys was prepared by mixing and homogenizing random quantities from each sample in a 250 mL beaker to simulate the batch collectors used in industry. Then, 8 g from each of the original honeydews and thyme honey, and 2 g from the batch flower honey, were transferred into a 50 mL beaker to prepare the 20% (*w*/*w*) adulterated samples. Finally, each sample was slightly heated at 37 °C, as previously mentioned, and subsequently submitted to ATR-FTIR analysis. Each sample was independently treated and analyzed in duplicate.

### 2.5. Statistical Analysis

The statistical processing of data involved pattern recognition and dimension reduction techniques [24]. In the first case, multivariate discriminant analysis, including multivariate analysis of variance and linear discriminant analysis, was applied to investigate the impact and classify samples according to botanical origin based on the transmittance values of the following ATR-FTIR spectral regions: Region A: 3300–3200 cm^−1^, Region B: 2970–2920 cm^−1^, Region C: 1730–1600 cm^−1^, Region D: 1420–1410 cm^−1^, Region E: 1390–1380 cm^−1^, Region F1: 1380–1330 cm^−1^, Region F2: 1380–1330 cm^−1^, Region G1: 1260–1225 cm^−1^, Region G2: 1260–1225 cm^−1^, Region H: 1210–1180 cm^−1^, Region I: 1150–1130 cm^−1^, Region J1: 1100–1010 cm^−1^, Region J2: 1100–1010 cm^−1^, Region J3: 1100–1010 cm^−1^, Region K1: 950–750 cm^−1^, Region K2: 950–750 cm^−1^, Region K3: 950–750 cm^−1^, and Region K4: 950–750 cm^−1^. The relocation of peaks in flower/polyfloral compared to fir, thyme, and pine honeys motivated us to process these different regional spectra, as valuable information could be collected regarding the botanical origin of honey samples. In the second case, factor analysis with principal component analysis as the extraction method was applied to identify the most critical spectral regions of ATR-FTIR related to the botanical origin of honey. Statistical analysis was done using the SPSS software v. 28.0 (IBM Inc., 2021, Armonk, NY, USA).

## 3. Results

### 3.1. Melissopalynological Analysis

Supplementary material, and especially Supplementary Tables S1–S59, include the analytical melissopalynological analysis results of all the studied honey samples. Tables S1–S16 represent the flower/polyfloral honey samples. Similarly, Tables S17–S30 represent the fir honey samples. Tables S31–S45 represent the thyme honey samples. Finally, Tables S46–S59 represent the pine honey samples.

### 3.2. ATR-FTIR Analysis

**Figure 1:**
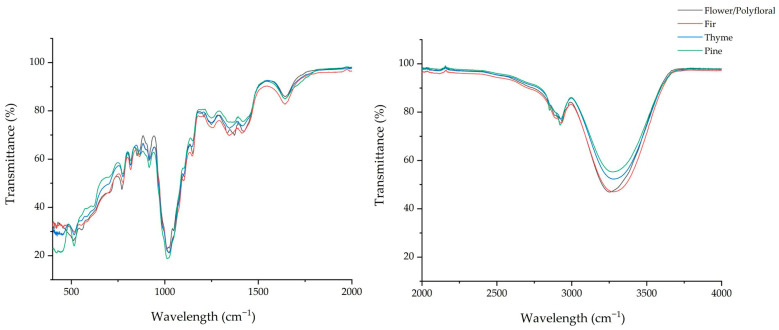
ATR-FTIR spectra of pure honey samples.

**Figure 2:**
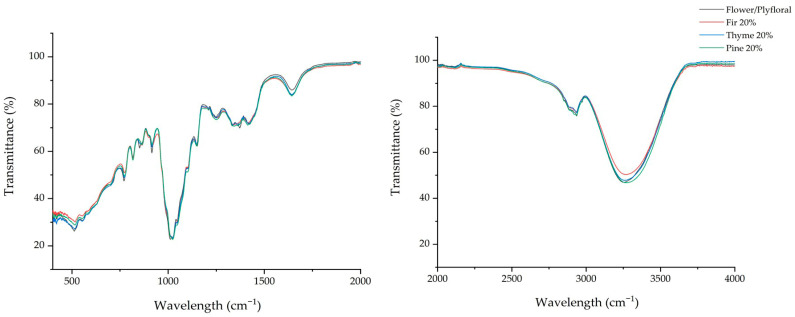
ATR-FTIR spectra of flower/polyfloral and adulterated honey samples.

Table 2 lists the transmittance values of the spectral regions under study for flower/polyfloral, fir, thyme, and pine honey samples and samples adulterated with the batch of floral/polyfloral honey at a concentration of 20% (*w*/*w*), indicating the presence of specific chemical classes such as water, carbohydrates, ketones, organic acids, and amino acids. Significant (*p* < 0.05) differences were recorded in all cases in the transmittance values of the monofloral, flower/polyfloral, and adulterated honey samples. As we mentioned previously in the Statistical Analysis section, the obtained infrared spectra were separated into 18 different wavenumber regions, including the regions of relocation of bonds owed to the specific honey type, in accordance with the current literature [20]. At the 3300–3200 cm^−1^ range, all the samples showed a broad peak due to their water content (O−H stretching and bending vibration) [20]. Flower/polyfloral honey showed a peak at 3252 cm^−1^, while the other honey types had their peak between 3271 and 3281 cm^−1^. At this point, we should stress that it is quite remarkable that when the pure honey types (fir, thyme, and pine) were adulterated with 20% flower/polyfloral honey, their peaks were slightly moved (relocation) to the pure flower/polyfloral peak (Figure 1 and Figure 2).

In the next wavenumber region (2970–2920), there is a peak that has been characterized in previous studies as assigned to carbohydrates, due to the C−H asymmetric and symmetric stretching vibrations [7,20]. Flower/polyfloral honey had a peak at 2936 cm^−1^, while the peaks of pure fir, thyme, and pine honeys were shifted within this region (2930 cm^−1^, 2927 cm^−1^, and 2923 cm^−1^, respectively). As previously mentioned, we can also clearly see here a displacement of the adulterated honeys toward the peak of the pure floral/polyfloral honey. This is a strong indication that when monofloral honeys are adulterated, their ATR-FTIR peaks shift significantly.

**Figure 3:**
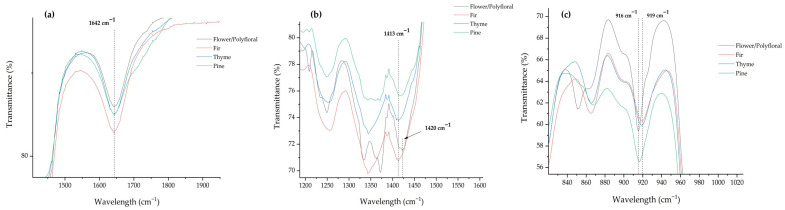
Peaks of pure monofloral honeys emerged as classification markers: (**a**) 1642 cm^−1^, (**b**) 1413 cm^−1^, and (**c**) 916 cm^−1^.

At the range of 1730–1600 cm^−1^, these shifts correspond to amino acids, organic acids, and carbohydrates, because of the N−H bending, C=O stretching, and H-O-H stretching [20,25]. The monofloral and adulterated honeys demonstrate a peak at 1642 cm^−1^, in agreement with previous studies [7,25]. However, the monofloral and adulterated honeys recorded statistically significant (*p* < 0.05) differences concerning their transmittance values, thus supporting their classification. Moreover, this peak may comprise a classification marker of pure honey types (Figure 3a).

Following the analysis, four different peaks were identified between 1420 and 1330 cm^−1^ in the majority of the samples, corresponding to the C−H rocking and O−H bending of carbohydrates. At the range of 1420–1410 cm^−1^, only the pure flower/polyfloral honey recorded a band at 1420 cm^—1^, while all the other samples had their bands at 1413 cm^—1^. Ioannou et al. [21] reported that the peak at 1420–1406 cm^−1^ was characteristic of honeys richer in monosaccharides, such as thyme, strawberry, and cotton, but was not present in honeydew honey. This was not the case in our study, as pine and fir honey types represented a peak in this area. Continuously, this area may comprise a classification marker of monofloral honey types (Figure 3b), indicating the importance of this wavenumber range. Additionally, as can be clearly seen in Table 2, all the honey samples recorded a peak at 1343–1344 cm^−1^, except for the flower/polyfloral and the adulterated thyme honey, which had their peaks at 1334–1335 cm^−1^. This is another example of peak displacement, when the monofloral honeys were adulterated with 20% (*w*/*w*) of flower/polyfloral honey.

Moving across the spectra, in the range of 1260–1225 cm^−1^ where the C–O and C–C stretching vibration is recorded, there is a remarkable and important observation. The pure flower/polyfloral honey had a band at 1234 cm^−1^, while none of the rest of the monofloral honeys (fir, thyme, and pine) had a peak in this area. Remarkably, when these honey types were adulterated, they represented a peak at 1234–1235 cm^−1^. This phenomenon can be attributed to the monosaccharides that are in higher concentration at the flower/polyfloral honey [26], and when it is added to pure honeydew and thyme honey, the monosaccharide content is increased, resulting in a sharp signal.

Another example of peak displacement after the adulteration was recorded at the wavenumber range of 1210–1180 cm^−1^. Pure fir, thyme, and pine honeys recorded peaks at 1193, 1191, and 1195 cm^−1^, respectively, while flower/polyfloral had its peak at 1209 cm^−1^. When the monofloral fir, thyme, and pine honeys were adulterated, their peaks were displaced at 1209 cm^−1^. This may be attributed to the monosaccharide content of flower/polyfloral honey, as well [26].

**Figure 4:**
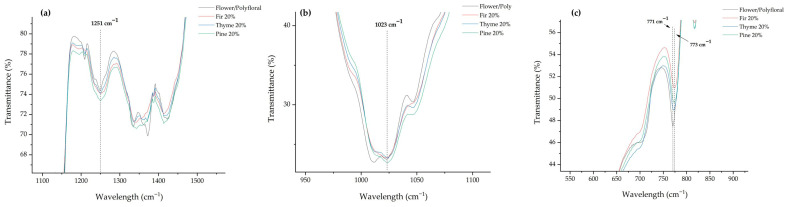
Peaks of the adulterated monofloral honeys emerged as classification markers: (**a**) 1251 cm^−1^, (**b**) 1023 cm^−1^, and (**c**) 771 cm^−1^.

One of the most important wavenumber ranges is 1100–1010 cm^−1^, corresponding to sucrose, glucose, and fructose [20]. All the monofloral and adulterated honeys represented their bands at 1023 cm^−1^, where the characteristic C–C stretching, C–O stretching, and C–H stretching vibration of glucose are obtained [20]. Moreover, this region emerged as a classification marker of the adulterated honey samples (Figure 4b). The fructose, flower/polyfloral honey recorded its band at 1047 cm^−1^, while none of the other pure honeys (fir, thyme, and pine) had any band in this area (Table 2). Again, when these honey types were adulterated, the sharp band of fructose was represented in the spectra, at the same wavenumber (1047 cm^−1^). This is attributed to the higher fructose content of flower/polyfloral honey compared to that of honeydew and thyme honey [27,28], resulting in the band appearance after their adulteration. Xagoraris et al. [7] also reported a band in this area (1044 cm^−1^) concerning Greek honeys.

Finally, in the region between 950 and 750 cm^−1^, the anomeric region of carbohydrates, four different bands were observed. Firstly, all the samples presented a band between 916 and 919 cm^−1^, indicating the presence of the β-configuration of anomeric carbon [29]. Next, flower/polyfloral honey had a band at 851 cm^−1^, while all the other samples had their bands between 867 and 864 cm^−1^. In addition, all the samples had a band at exactly 817 cm^−1^. This peak was attributed to fructose [21]. Lastly, at the 770–780 cm^−1^ region, the flower/polyfloral honey recorded a band at 771 cm^−1^, while the pure honey samples (fir, thyme, and pine) had their bands at 776 cm^−1^. Peak in this area indicates the presence of a pyran ring [29]. When the monofloral honeys were adulterated, their peaks were slightly displaced near the pure flower/polyfloral honey band, which is another strong indication of awareness. In a previous study, Ioannou et al. [21] reported significant peaks in different honey types (i.e., oak, thyme, fin, blossom, etc.) from northern Greece at the same regions, while Xagoraris et al. [7] obtained bands at the same wavenumber in thyme, pine, and fir honey samples from Greece.

Before going any further and to support the hypothesis driven in the study, we should keep in mind that beekeepers are shifting from using syrups and beecakes to polyfloral honey for adulteration. This highlights a concerning trend: adulteration techniques are evolving and becoming more sophisticated [30,31]. For example, the use of polyfloral honey to adulterate monofloral honey is particularly challenging to detect because it mimics natural composition (polyfloral honey can have a more similar composition to monofloral honey, making it harder to detect adulteration); unlike syrups or sugar solutions [22,30], polyfloral honey does not clearly introduce obvious markers (e.g., sugar profiles) that can be easily detected. These limitations may probably be surpassed when the data are treated with multivariate statistics [13,21,31]. Finally, different levels (*w*/*w*) of honey falsification could strengthen further the results of the present study and the applicability of the methodological procedure, although in the literature, the falsification level of higher than 10% (*w*/*w*) is recommended for the robust adulteration testing of monofloral honey using more sophisticated techniques such as nuclear magnetic resonance [31].

### 3.3. Βotanical Origin Confirmation of Monofloral Honey Using ATR-FTIR

#### 3.3.1. Multivariate Analysis of Variance (MANOVA)

The qualitative criteria of the multivariate hypothesis, Pillai’s Trace = 2.870 (F = 49.156, df = 54, *p* < 0.001), and Wilks’ Lambda = 0.000 (F = 92.743, df = 54, *p* < 0.001), both having an observed power of 1.000, showed that there was a significant impact (*p* < 0.05) of the botanical origin of honey on the majority of the transmittance values of the studied spectral regions during ATR-FTIR analysis (Table S60).

#### 3.3.2. Factor Analysis (FA)

Factor analysis showed that the spectral regions of the different honey types under investigation describe the variability in the multidimensional space. The Keiser–Meyer–Olkin (KMO) index was 0.692, while Bartlett’s Test of Sphericity yielded Chi-square (X^2^) = 2526.033, df = 136, *p* < 0.001, indicating the presence of correlations among the transmittance values of the studied spectral regions during ATR-FTIR analysis, in relation to the botanical origin of honey, thereby allowing the effective application of factor analysis.

The principal components (PCs) that showed the highest correlation (factors). Based on the first three PCs, the variance explained was 90.31%, which is considered very good. The transmittance values of the spectral regions for which the correlation in the rotated component matrix of the multidimensional space was the largest were those in 1420–1410 cm^−1^ (PC1, correlation of 0.989 and explained variance of 55.29%), 950–750 cm^−1^ (PC2, correlation of 0.901 and explained variance of 18.41%), and 1730–1600 cm^−1^ (PC3, correlation of 0.805 and explained variance of 16.61%).

#### 3.3.3. Linear Discriminant Analysis (LDA)

Three discriminant functions were derived in LDA to differentiate honey samples by botanical origin using the significant (*p* < 0.05) transmittance values of the spectral regions under study. The analytical data of these discriminant functions, including the Wilks’ Lambda, Chi-square, eigenvalues, explained and cumulative variance, along with canonical correlation, are given in Table 3.

**Figure 5:**
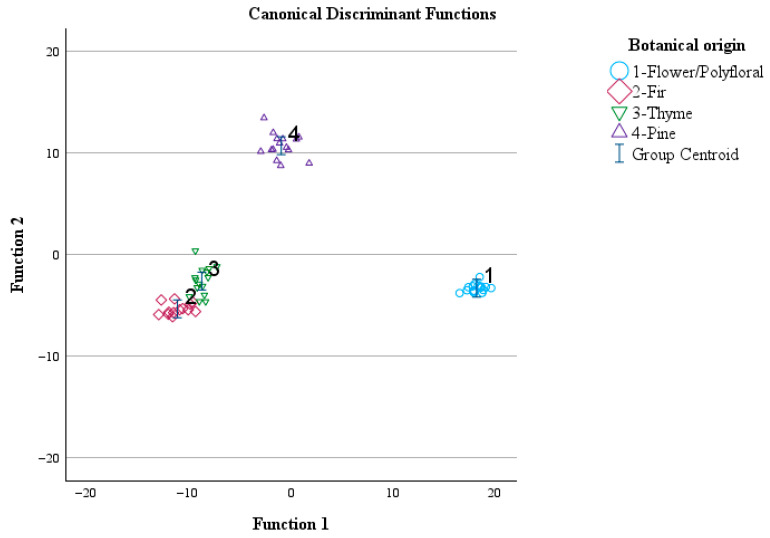
Classification of monofloral and flower/polyfloral honey according to botanical origin using the transmittance values of the spectral regions during ATR-FTIR analysis and LDA. Function 1: discriminant function 1. Function 2: discriminant function 2.

The overall correct classification rate was 100% for the original and 100% for the cross-validation method. The group centroid values were (18.268, −3.288), (−11.201, −5.328), (−8.802, −2.592), and (−0.976, 10.717) for flower, fir, thyme, and pine honey samples, respectively (Figure 5).

The transmittance values of the spectral regions that contributed most to the confirmation of the botanical origin of honey samples were those with the highest absolute correlation values within the discriminant functions and are summarized in bold in Table 4.

### 3.4. Βotanical Origin Adulteration Testing of Monofloral Honey Using ATR-FTIR

#### 3.4.1. MANOVA

In this section, fir, thyme, and pine honey samples were adulterated with flower/polyfloral honey (20% *w*/*w*). Pillai’s Trace = 4.449 (F = 13.232, df = 108, *p* < 0.001) and Wilks’ Lambda = 0.000 (F = 34.427, df = 108, *p* < 0.001), both having an observed power of 1.000, showed that there was a significant impact of the botanical origin of honey on the transmittance values of the spectral regions during ATR-FTIR analysis (Table S61).

#### 3.4.2. FA

Factor analysis showed that the transmittance values of the spectral regions of the different honey types under investigation describe the variability in the multidimensional space. The Keiser–Meyer–Olkin (KMO) index was 0.753, while Bartlett’s Test of Sphericity yielded Chi-square (X^2^) = 3910.432, df = 136, *p* < 0.001, indicating the presence of correlations among the transmittance values of the spectral regions during ATR-FTIR analysis in relation to the botanical origin of honey, thereby allowing again, the effective application of factor analysis. The principal components (PCs) that showed the highest correlation (factors) were three. Based on the first three PCs, the variance explained was 87.13%, considered very good. The transmittance values of the spectral regions for which the correlation in the rotated component matrix of the multidimensional space was the largest were those in 950–750 cm^−1^ (PC1, correlation of 0.959 and explained variance of 35.04%), 1260–1225 cm^−1^ (PC2, correlation of 0.889 and explained variance of 34.77%), and 1150–1130 cm^−1^ (PC3, correlation of 0.895 and explained variance of 17.32%).

#### 3.4.3. LDA

The results of LDA in this case showed that six discriminant functions were formed. Similarly, the analytical data of these discriminant functions are given in Table 5. The overall correct classification rate was 97.1% for the original and 91.3% for the cross-validation method. Between these values, the latter is considered very satisfactory, given that the error in the classification of honey samples was below 9%.

**Figure 6:**
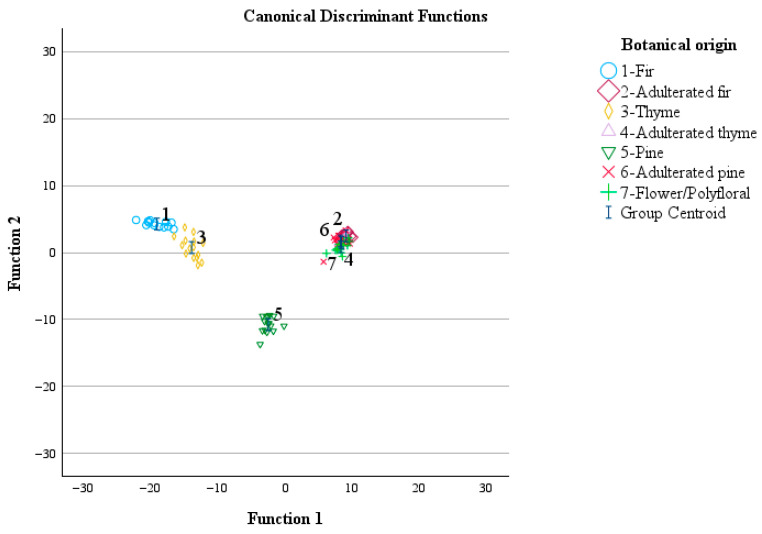
Classification of monofloral and adulterated honey samples with flower/polyfloral honey according to botanical origin using the transmittance values of the spectral regions during ATR-FTIR analysis and LDA.

The group centroid values were (−19.195, 4.268), (9.044, 2.487), (−14.018, 0.745), (8.330, 1.497), (−2.568, −10.750), (8.231, 1.736), and (8.272, 0.860) for fir, fir adulterated with 20% (*w*/*w*) of flower/polyfloral honey, thyme, thyme adulterated with 20% (*w*/*w*) of flower/polyfloral honey, pine, pine adulterated with 20% (*w*/*w*) of flower/polyfloral honey, and flower/polyfloral honey, respectively (Figure 6).

Perfect classification (100%) based on the cross-validation method was obtained for fir, thyme, and pine honey samples, whereas the respective classification performance for the other samples categories was flower/polyfloral honey (93.8%), fir honey adulterated with 20% (*w*/*w*) of flower/polyfloral honey (92.3%), thyme honey adulterated with 20% (*w*/*w*) of flower/polyfloral honey (80%), and pine honey adulterated with 20% (*w*/*w*) of flower/polyfloral honey (73.3%).

The transmittance values of the spectral regions that contributed most to the discrimination of honey samples of different botanical origin were those pooled with the highest absolute correlation value (with bold lettering) within the discriminant functions (Table 6).

## 4. Conclusions

Results of the present study showed that ATR-FTIR can be considered as an emerging analytical technique to assess the authenticity and adulteration testing of Greek monofloral and polyfloral honey. The results of ATR-FTIR analysis fully confirmed the melissopalynological analysis results in combination with pattern recognition and dimension reduction techniques. To date, limited studies are available in the literature reporting analytical ATR-FTIR data for Greek honey, whereas scarce data are available in the literature regarding the adulteration of monofloral honey using other types of flower/polyfloral honey, which constitutes the novelty of the present study, while supporting previous studies in the literature. At the same time, new perspectives for the use of green and rapid methods of analysis for the botanical origin authentication and adulteration control of Greek honey are being developed.

## Figures and Tables

**Table 1:** Sample number, botanical origin, geographical origin, and geographical coordinates of the honey samples.

Honey Sample Number as Provided by ATTIKI Bee Co.	Botanical Origin	Geographical Region	Coordinates
1	Orange	Argos	37°38′0″ N, 22°43′45″ E
2	Orange	Argos	37°38′0″ N, 22°43′45″ E
3	Flower/Asfaka	Itea/Galaksidi	38°24′15.9″ N, 22°24′27.0″ E
4	Flower/Thyme	Galaksidi	38°22′ 39.0″ N, 22°22′58.8″ E
5	Asfaka	Galaksidi	38° 22′ 39.0″ N, 22°22′58.8″ E
6	Orange	Arta	39°9′29.52″ N, 20°59′15.72″ E
7	Flower	Evros	41°34′12″ N, 26°37′48″ E
9	Flower	Chania	35°30′40.32″ N, 24°1′45.12″ E
10	Flower/Thyme	Chania	35°30′40.32″ N, 24°1′45.12″ E
11	Asfaka	Itea/Galaksidi	38°24′15.9″ N, 22°24′27.0″ E
13	Flower	Karditsa	39°21′53″ N, 21°55′19″ E
14	Fir	Karditsa	39°21′53″ N, 21°55′19″ E
16	Flower	Heraklion	35°20′19.32″ N, 25°8′39.12″ E
18	Thyme	Chania	35°30′40.32″ N, 24°1′45.12″ E
19	Thyme	Chania	35°30′40.32″ N, 24°1′45.12″ E
20	Thyme	Chania	35°30′40.32″ N, 24°1′45.12″ E
21	Fir	Parnonas	37°6′21.6″ N, 22°43′48″ E
22	Flower	Thessaly	39°30′0″ N, 22°0′0″ E
23	Fir	Karpenisi	38°54′43.92″ N, 21°47′54.24″ E
24	Fir	Karpenisi	38°54′43.92″ N, 21°47′54.24″ E
25	Pine	Chalkidiki	40°16′0.12″ N, 23°30′0.0″ E
27	Flower/Thyme	Chania	35°30′40.32″ N, 24°1′45.12″ E
28	Thyme	Chania	35°30′40.32″ N, 24°1′45.12″ E
29	Thyme	Chania	35°30′40.32″ N, 24°1′45.12″ E
31	Thyme	Chania	35°30′40.32″ N, 24°1′45.12″ E
32	Flower	Chania	35°30′40.32″ N, 24°1′45.12″ E
33	Flower	Itea/Galaksidi	38°24′15.9″ N, 22°24′27.0″ E
34	Pine	Thasos	40°41′2.93″ N, 24°39′39.9″ E
35	Pine	Thasos	40°41′2.93″ N, 24°39′39.9″ E
36	Pine	Thasos	40°41′2.93″ N, 24°39′39.9″ E
37	Pine	Thasos	40°41′2.93″ N, 24°39′39.9″ E
38	Pine	Thasos	40°41′2.93″ N, 24°39′39.9″ E
39	Pine	Thasos	40°41′2.93″ N, 24°39′39.9″ E
40	Pine	Thasos	40°41′2.93″ N, 24°39′39.9″ E
41	Pine	Thasos	40°41′2.93″ N, 24°39′39.9″ E
42	Pine	Thasos	40°41′2.93″ N, 24°39′39.9″ E
43	Thyme	Chania	35°30′40.32″ N, 24°1′45.12″ E
44	Pine	Tripoli	37°30′32.04″ N, 22°22′44.4″ E
45	Thyme	Chania	35°30′40.32″ N, 24°1′45.12″ E
46	Thyme	Chania	35°30′40.32″ N, 24°1′45.12″ E
47	Fir	Karpenisi	38°54′43.92″ N, 21° 47′54.24″ E
48	Pine	Chalkidiki	40°16′0.12″ N, 23°30′0.0″ E
49	Pine	Chalkidiki	40°16′0.12″ N, 23°30′0.0″ E
50	Pine	Chalkidiki	40°16′0.12″ N, 23°30′0.0″ E
51	Fir	Vitina	37°40′0.12″ N, 22°12′0.0″ E
52	Thyme	Chania	35°30′40.32″ N, 24°1′45.12″ E
53	Thyme	Chania	35°30′40.32″ N, 24°1′45.12″ E
54	Fir	Karpenisi	38°54′43.92″ N, 21°47′54.24″ E
55	Pine	Evia	38°30′0″ N, 24°0′0″ E
57	Fir	Karpenisi	38°54′43.92″ N, 21°47′54.24″ E
58	Thyme	Kos	36°48′ 55.08″ N, 27°6′37.08″ E
59	Thyme	Kos	36°48′ 55.08″ N, 27°6′37.08″ E
60	Fir	Parnonas	37°6′21.6″ N, 22°43′48″ E
61	Pine	Tripoli	37°30′32.04″ N, 22°22′44.4″ E
62	Thyme	Chania	35°30′40.32″ N, 24°1′45.12″ E
63	Fir	Parnonas	37°6′21.6″ N, 22°43′48″ E
64	Fir	Parnonas	37°6′21.6″ N, 22°43′48″ E
65	Fir	Karpenisi	38°54′43.92″ N, 21°47′54.24″ E
67	Thyme	Chania	35°30′40.32″ N, 24°1′45.12″ E

**Table 2:** Chemical classes, wavenumber (cm^−1^), and transmittance values (T%) of monofloral and adulterated honey samples during ATR-FTIR analysis.

Wavenumber (cm^−1^)	Group	Compounds	Floral/Polyfloral Peaks (cm^−1^)	Flower/Polyfloral T (%)	Fir Peaks (cm^−1^)	Fir T (%)	Adult. Fir Peaks (cm^−1^)	Adult. Fir T (%)	Thyme Peaks (cm^−1^)	Thyme T (%)	Adult. Thyme Peaks (cm^−1^)	Adult. Thyme T (%)	Pine Peaks (cm^−1^)	Pine T (%)	Adult. Pine Peaks (cm^−1^)	Adult. Pine T (%)
3300–3200	O–H stretching and bending vibration	water, carbohydrates, organic acids	3252	46.95 ± 2.69 ^aA^	3282	47.04 ± 0.70 ^aA^	3271	50.31 ± 0.77 ^B^	3281	52.32 ± 2.48 ^bCB^	3254	47.86 ± 2.25 ^A^	3274	55.28 ± 3.09 ^cD^	3272	46.78 ± 1.30 ^A^
2970–2920	C–H asymmetrical and symmetrical stretching vibration	carbohydrates, carboxylic acids, amino acids	2936	75.76 ± 1.64 ^aA^	2930	75.47 ± 1.28 ^aA^	2934	77.71 ± 0.59 ^Β^	2927	77.24 ± 1.97 ^aAΒ^	2936	77.17 ± 1.52 ^AΒ^	2923	74.68 ± 2.39 ^abA^	2935	76.13 ± 0.42 ^AΒ^
1730–1600 *	O–H stretching and bending, C=O stretching, N–H bending	water, carbohydrates, ketones, amino acids	1642	85.89 ± 0.94 ^aA^	1642	82.70 ± 0.94 ^bΒ^	1642	83.53 ± 0.71 ^Β^	1642	84.93 ± 0.49 ^cC^	1642	83.76 ± 0.73 ^B^	1642	84.89 ± 0.93 ^cC^	1642	83.37 ± 0.56 ^B^
1420–1410 *	O–H bending, C–H rocking	carbohydrates, organic acids	1420	71.53 ± 1.61 ^aA^	1413	70.71 ± 0.66 ^aA^	1413	72.05 ± 0.54 ^AB^	1413	73.73 ± 1.08 ^bC^	1413	71.89 ± 1.02 ^A^	1413	75.68 ± 1.49 ^cD^	1413	71.11 ± 0.62 ^A^
1390–1380	O–H bending, C–H rocking	carbohydrates, organic acids	1387	74.34 ± 1.39 ^aA^	1388	72.63 ± 0.72 ^bB^	1387	73.80 ± 0.52 ^A^	1388	75.39 ± 0.91 ^aCA^	1387	73.83 ± 0.98 ^A^	1387	77.01 ± 1.29 ^cD^	1387	73.18 ± 0.55 ^A^
1380–1330	O–H and C–H bending	carbohydrates	1371	69.87 ± 2.80 ^aA^	-	71.54 ± 0.68 ^aA^	1371	72.25 ± 0.62 ^AB^	-	74.41 ± 0.91 ^bC^	1371	71.38 ± 1.23 ^A^	1371	75.29 ± 2.19 ^bC^	1371	71.30 ± 0.73 ^A^
			1334	70.83 ± 1.57 ^aA^	1343	69.79 ± 0.65 ^aA^	1343	71.23 ± 0.67 ^BA^	1344	72.78 ± 1.08 ^bC^	1335	71.27 ± 1.01 ^B^	1343	75.30 ± 1.48 ^cB^	1343	70.61 ± 0.65 ^A^
1260–1225	C–O and C–C stretching vibration	carbohydrates	1249	74.37 ± 1.35 ^aA^	1256	70.39 ± 0.65 ^bB^	1251	74.08 ± 0.57 ^A^	1252	73.22 ± 1.02 ^cA^	1251	74.12 ± 0.92 ^A^	1249	77.08 ± 1.06 ^dD^	1251	73.39 ± 0.64 ^AE^
			1234	75.53 ± 1.39 ^aA^	-	74.09 ± 0.71 ^bB^	1235	74.87 ± 0.60 ^B^	-	75.96 ± 0.82 ^aA^	1235	75.02 ± 0.93 ^AB^	-	77.94 ± 0.96 ^cC^	1235	74.27 ± 0.67 ^CD^
1210–1180	C–C ring stretching vibration	carbohydrates	1209	77.46 ± 1.68 ^aA^	1193	77.57 ± 0.72 ^aA^	1209	78.37 ± 0.46 ^A^	1191	79.01 ± 0.67 ^bBA^	1209	78.01 ± 0.84 ^A^	1195	80.41 ± 0.47 ^cC^	1209	77.85 ± 0.53 ^A^
1150–1130	C–H deformation, C–O stretching vibration	carbohydrates	1150	62.17 ± 2.01 ^aA^	1146	61.27 ± 0.78 ^aA^	1147	63.08 ± 0.53 ^B^	1144	65.13 ± 1.19 ^bC^	1148	63.09 ± 1.17 ^B^	1147	67.61 ± 2.28 ^cD^	1148	62.31 ± 0.41 ^AB^
1100–1010	C–C stretching, C–O stretching, C–H stretching	carbohydrates, organic acids	1011	22.72 ± 3.30 ^aA^	-	22.64 ± 0.94 ^aA^	-	24.32 ± 0.94 ^AB^	-	22.07 ± 1.75 ^aA^	-	24.15 ± 1.17 ^A^	1011	18.71 ± 2.22 ^bC^	-	23.45 ± 1.63 ^A^
			1023	22.72 ± 3.30 ^aA^	1023	28.11 ± 0.53 ^bB^	1023	23.29 ± 0.69 ^A^	1023	27.77 ± 1.43 ^bB^	1023	23.30 ± 0.94 ^A^	1023	27.17 ± 1.76 ^bB^	1023	22.71 ± 1.45 ^A^
			1047	30.38 ± 2.81 ^aA^	-	28.11 ± 0.54 ^bB^	1047	30.26 ± 0.59 ^A^	-	27.77 ± 1.44 ^bB^	1047	29.64 ± 1.21 ^BA^	-	27.02 ± 1.68 ^bB^	1047	28.68 ± 1.31 ^BA^
950–750 *	C–H bending vibration (anomeric region)	carbohydrates	916	59.69 ± 3.42 ^aA^	919	60.30 ± 1.88 ^aA^	916	62.10 ± 0.59 ^A^	919	59.92 ± 2.08 ^aA^	916	61.89 ± 1.32 ^A^	916	56.53 ± 2.93 ^bB^	916	62.84 ± 1.21 ^BA^
			851	61.46 ± 4.20 ^aA^	865	61.02 ± 1.09 ^aA^	864	62.59 ± 0.74 ^A^	865	61.96 ± 0.86 ^aA^	864	62.85 ± 1.06 ^A^	867	61.81 ± 1.37 ^aA^	864	62.87 ± 0.93 ^A^
			817	57.49 ± 1.85 ^aA^	817	55.66 ± 0.67 ^bB^	817	56.89 ± 0.61 ^BA^	817	57.58 ± 0.81 ^aAB^	817	56.44 ± 1.01 ^B^	817	59.38 ± 0.79 ^cC^	817	56.35 ± 1.28 ^B^
			771	47.60 ± 4.00 ^aA^	776	50.39 ± 0.49 ^bB^	773	50.97 ± 0.68 ^B^	776	52.72 ± 0.97 ^bC^	772	48.95 ± 1.51 ^BA^	776	53.93 ± 1.90 ^cC^	773	49.62 ± 1.52 ^A^

Different letters in each line (a–d) indicate statistically significant (*p* < 0.05) differences between the pure honey types. Different letters in each line (A–E) indicate statistically significant (*p* < 0.05) differences between the pure and adulterated honey types. * Key spectral regions of interest.

**Table 3:** Analytical data of LDA for the botanical origin confirmation of monofloral and flower/polyfloral honey.

Test of Function(s)	Wilks’ Lambda	Chi-Square	df	*p*-Value	Eigenvalue	% of Variance	Cumulative %	Canonical Correlation
1 through 3	0.000	524.326	51	<0.001	148.124 ^a^	74.2	74.2	0.997
2 through 3	0.002	286.599	32	<0.001	43.012 ^a^	21.5	95.8	0.989
3	0.105	106.837	15	<0.001	8.480 ^a^	4.2	100.0	0.946

^a^ First 3 canonical discriminant functions were used in the analysis.

**Table 4:** Contribution of the transmittance values of the spectral regions during ATR-FTIR analysis for the botanical origin confirmation of monofloral and flower/polyfloral honey using LDA.

Structure Matrix			
Spectral Regions (cm^−1^) (Transmittance Values)	Function		
	1	2	3
Region J2 (1100–1010)	**−0.089** *	0.030	0.004
Region G1 (1260–1225)	0.080	**0.299** *	−0.204
Region F2 (1380–1330)	−0.017	**0.237** *	−0.215
Region G2 (1260–1225)	0.014	**0.195** *	−0.156
Region H (1210–1180)	−0.031	**0.167** *	−0.126
Region K3 (950–750)	0.024	**0.155** *	−0.144
Region J1 (1150–1130)	0.008	**−0.114** *	−0.005
Region K4 (950–750)	−0.061	**0.112** *	−0.102
Region K1 (950–750)	−0.003	**−0.088** *	−0.013
Region J3 (1100–1010)	0.048	**−0.064** *	0.016
Region C (1730–1600)	0.088	0.049	**−0.284** *
Region E (1390–1380)	0.008	0.190	**−0.237** *
Region D (1420–1410)	−0.022	0.209	**−0.225** *
Region I (1150–1130)	−0.024	0.203	**−0.219** *
Region A (3300–3200)	−0.042	0.193	**−0.212** *
Region F1 (1380–1330)	−0.059	0.128	**−0.160** *
Region B (2970–2920)	−0.009	−0.047	**−0.135** *

Pooled within-groups correlations between discriminating variables and standardized canonical discriminant functions. Variables ordered by the absolute size of correlation within the function. * Largest absolute correlation between each variable and any discriminant function.

**Table 5:** Analytical data of LDA for the botanical origin confirmation of monofloral honey adulterated with flower/polyfloral honey using LDA.

Test of Function(s)	Wilks’ Lambda	Chi-Square	df	*p*-Value	Eigenvalue	% of Variance	Cumulative %	Canonical Correlation
1 through 6	0.000	1079.340	102	<0.001	126.858 ^a^	80.2	80.2	0.996
2 through 6	0.001	647.608	80	<0.001	22.628 ^a^	14.3	94.5	0.979
3 through 6	0.016	366.152	60	<0.001	5.190 ^a^	3.3	97.7	0.916
4 through 6	0.101	203.913	42	<0.001	1.809 ^a^	1.1	98.9	0.802
5 through 6	0.284	111.999	26	<0.001	1.027 ^a^	0.6	99.5	0.712
6	0.576	49.097	12	<0.001	0.736 ^a^	0.5	100.0	0.651

^a^. First 6 canonical discriminant functions were used in the analysis.

**Table 6:** Contribution of the transmittance values of the spectral regions during ATR-FTIR analysis for the botanical origin confirmation of monofloral honey adulterated with flower/polyfloral honey using LDA.

Structure Matrix						
Spectral Regions (cm^−1^) (Transmittance Values)	Function					
	1	2	3	4	5	6
Region J2 (1100–1010)	−0.117 *	−0.089	−0.026	0.078	0.112	−0.089
Region G1 (1260–1225)	0.084	−0.351 *	0.139	0.184	0.223	−0.053
Region E (1390–1380)	−0.014	−0.273 *	0.217	0.229	0.198	0.011
Region G2 (1260–1225)	−0.011	−0.268 *	0.169	0.083	0.233	−0.027
Region K3 (950–750)	0.002	−0.201 *	0.138	0.085	0.170	0.193
Region J1 (1150–1130)	0.033	0.178 *	−0.025	0.091	0.052	−0.164
Region C (1730–1600)	0.020	−0.130	0.506 *	−0.125	0.165	0.342
Region A (3300–3200)	−0.038	−0.251	0.031	0.493 *	0.394	0.010
Region F1 (1380–1330)	−0.049	−0.172	−0.030	0.475 *	0.095	−0.079
Region I (1150–1130)	−0.022	−0.279	0.082	0.422 *	0.117	−0.157
Region K4 (950–750)	−0.050	−0.144	−0.090	0.411 *	0.120	0.064
Region D (1420–1410)	−0.027	−0.286	0.113	0.378 *	0.219	−0.112
Region F2 (1380–1330)	−0.019	−0.313	0.093	0.355 *	0.145	−0.125
Region H (1210–1180)	−0.023	−0.211	−0.024	0.332 *	0.125	−0.062
Region B (2970–2920)	0.013	0.083	0.046	0.328 *	0.178	−0.189
Region K1 (950–750)	0.033	0.162	−0.092	0.190 *	−0.189	−0.094
Region J3 (1100–1010)	0.049	0.095	0.061	−0.109	0.274 *	0.024

Pooled within-groups correlations between discriminating variables and standardized canonical discriminant functions. Variables are ordered by the absolute size of correlation within the function. * Largest absolute correlation between each variable and any discriminant function.

## Data Availability

The original contributions presented in this study are included in the article/Supplementary Materials. Further inquiries can be directed to the corresponding author.

## Supplementary Materials

The following supporting information can be downloaded at: https://www.mdpi.com/article/10.3390/foods15091544/s1, Tables S1–S59: Analytical Melissoplaynological analysis results, Tables S60 and S61: MANOVA analysis results.
