# Supplementary material for: Βotanical Origin Confirmation and Adulteration Testing of Monofloral Honey Using ATR-FTIR Spectroscopy in Combination with Pattern Recognition and Dimension Reduction Techniques
Source: Foods. 2026 Apr 29;15(9):1544. doi: 10.3390/foods15091544 (PMC13163368; doi:10.3390/foods15091544)
Supplement: Supplementary file 1 [file foods-15-01544-s001.zip › foods-4256891-supplementary.pdf]

<u>**Supplementary Material for Online Review**</u>

**Tables S1 to S59:** Analytical Melissopalynological Analysis Results

✓ **Flower/Polyfloral honeys**

**Table S1.** Sample no. 1: Citrus honey from Argos.

| | |
|---|---|
| Predominant pollen (>45%) | |
| Secondary pollen (16-45%) | Liliaceae (28%),<br>*Brassica* sp. Brassicaceae (16%) |
| Important minor pollen (3-15%) | *Hedera helix* Araliaceae (13%),<br>*Citrus* sp. Rutaceae (9%),<br>*Rubus* sp. Rosaceae (9%),<br>*Castanea sativa* Fagaceae (6%),<br>*Polygonum aviculare* Polygonaceae (5%),<br>*Erica* sp. Ericaceae (3%),<br>Rosaceae (3%),<br>Asteraceae (3%),<br>*Vicia* sp. Fabaceae (3%) |
| Minor pollen ( <3%) | |
| Pollen from nectarless plants/Isolated pollen | *Cistus* sp. Cistaceae,<br>*Helianthemum* sp. Cistaceae,<br>*Ephedra* sp. Ephedraceae,<br>*Hypericum* sp. Hypericaceae,<br>*Olea europaea* Oleaceae,<br>Poaceae,<br>Apiaceae,<br>*Robinia pseudacacia* Fabaceaea,<br>*Ashodelus* sp. Liliaceae |

| | |
|---|---|
| | |

**Table S2.** Samples no.2: Citrus honey from Argos.

| | |
|---|---|
| Predominant pollen (>45%) | |
| Secondary pollen (16-45%) | *Trifolium* sp. Fabaceae (25%),<br>*Hedera helix* Araliaceae (25%),<br>*Carthamus lanatus* Asteraceae (18%) |
| Important minor pollen (3-15%) | Asteraceae (13%),<br>*Citrus* sp. Rutaceae (7%),<br>*Brassica* sp. Brassicaceae (7%),<br>*Paliurus spina-christi* Rhamnaceae (3%) |
| Minor pollen ( <3%) | |
| Pollen from nectarless plants/Isolated pollen | *Helianthemum* sp. Cistaceae,<br>*Hypericum* sp. Hypericaceae,<br>*Olea europaea* Oleaceae,<br>Caprifoliaceae,<br>*Vicia* sp. Fabaceae,<br>Boraginaceae,<br>Rosaceae,<br>Liliaceae,<br>*Robinia pseudacacia* Fabaceaea |

**Table S3.** Sample no.3: Flower/Asfaka honey from Itea-Galaxidi.

| | |
|---|---|
| Predominant pollen (>45%) | *Trifolium* sp. Fabaceae (64%) |
| Secondary pollen (16-45%) | |
| Important minor pollen (3-15%) | *Brassica* sp. Brassicaceae (14%),<br>*Phlomis Fruticosa* Lamiaceae (13%),<br>*Robinia pseudacacia* Fabaceaea (5%) |
| Minor pollen ( <3%) | Rosaceae (2%) |
| Pollen from nectarless plants/Isolated pollen | Lamiaceae (*Thymus* type),<br>Asteraceae,<br>*Olea europaea* Oleaceae,<br>Caprifoliaceae,<br>*Cistus* sp. Cistaceae,<br>*Hypericum* sp. Hypericaceae,<br>*Convolvulus arvensis* Convolvulaceae |

**Table S4.** Sample no. 4: Flower/Thyme honey from Galaxidi.

| | |
|---|---|
| Predominant pollen (>45%) | |
| Secondary pollen (16-45%) | |
| Important minor pollen (3-15%) | *Thymus* sp. Lamiaceae (13%),<br>*Trifolium* sp. Fabaceae (13%),<br>*Brassica* sp. Brassicaceae (13%),<br>Rosaceae (13%),<br>*Hedera helix* Araliaceae (13%),<br>*Polygonum aviculare* Polygonaceae (9%),<br>*Phlomis fruticosa* Lamiaceae (9%),<br>Liliaceae (9%),<br>*Citrus* sp. Rutaceae (4%) |
| Minor pollen ( <3%) | *Robinia pseudacacia* Fabaceaea (2%) |
| Pollen from nectarless plants/Isolated pollen | *Carthamus lanatus* Asteraceae,<br>Asteraceae,<br>*Ashodelus* sp. Liliaceae,<br>*Vicia* sp. Fabaceae,<br>Apiaceae,<br>*Erica* sp. Ericaceae,<br>*Olea europaea* Oleaceae,<br>*Cistus* sp. Cistaceae |

**Table S5.** Sample no. 5: Asfaka honey from Galaxidi.

| | |
|---|---|
| Predominant pollen (>45%) | *Trifolium* sp. Fabaceae (52%) |
| Secondary pollen (16-45%) | *Phlomis Fruticosa* Lamiaceae (21%) |
| Important minor pollen (3-15%) | Rosaceae (6%),<br>*Brassica* sp. Brassicaceae (6%),<br>*Paliurus spina-christi* Rhamnaceae (6%),<br>Pyrus/Prunus Rosaceae (4%),<br>Liliaceae (3%) |
| Minor pollen ( <3%) | |
| Pollen from nectarless plants/Isolated pollen | *Hedera helix* Araliaceae,<br>*Helianthemum* sp. Cistaceae,<br>*Hypericum* sp. Hypericaceae,<br>*Vicia* sp. Fabaceae,<br>Boraginaceae,<br>*Castanea sativa* Fagaceae,<br>*Quercus ilex* Fagaceae,<br>*Olea europaea* Oleaceae,<br>Poaceae,<br>*Convolvulus arvensis* Convolvulaceae,<br>*Cistus* sp. Cistaceae |

**Table S6.** Sample no.6: Citrus honey from Arta.

| | |
|---|---|
| Predominant pollen (>45%) | |
| Secondary pollen (16-45%) | *Phlomis Fruticosa* Lamiaceae (30%), *Trifolium* sp. Fabaceae (30%) |
| Important minor pollen (3-15%) | *Brassica* sp. Brassicaceae (10%),<br>Liliaceae (6%),<br>*Carthamus lanatus* Asteraceae (5%),<br>*Paliurus spina-christi* Rhamnaceae (5%),<br>*Vicia* sp. Fabaceae (4%),<br>Boraginaceae (4%)<br>*Citrus* sp. Rutaceae (4%), |
| Minor pollen ( <3%) | |
| Pollen from nectarless plants/Isolated pollen | *Olea europaea* Oleaceae,<br>*Helianthemum* sp. Cistaceae,<br>*Cistus* sp. Cistaceae,<br>Apiaceae,<br>*Rubus* sp. Rosaceae,<br>*Castanea sativa* Fagaceae,<br>*Quercus ilex* Fagaceae,<br>Myrtaceae |

**Table S7.** Sample no. 7: Flower honey from Evros.

| | |
|---|---|
| Predominant pollen (>45%) | |
| Secondary pollen (16-45%) | *Helianthus annuus* Asteraceae (31%),<br>*Brassica* sp. Brassicaceae (27%),<br>*Paliurus spina-christi* Rhamnaceae (23%) |
| Important minor pollen (3-15%) | *Castanea sativa* Fagaceae (13%) |
| Minor pollen ( <3%) | *Trifolium* sp. Fabaceae (2%),<br>Apiaceae (2%) |
| Pollen from nectarless plants/Isolated pollen | *Cistus* sp. Cistaceae,<br>*Hypericum* sp. Hypericaceae,<br>Poaceae,<br>Asteraceae,<br>*Erica* sp. Ericaceae<br>*Centaurea* sp. Asteraceae,<br>Chenopodiaceae |

**Table S8.** Sample no. 9: Flower honey from Hania (Crete).

| | |
|---|---|
| Predominant pollen (>45%) | |
| Secondary pollen (16-45%) | *Ballota* sp. Lamiaceae (36%),<br>*Castanea sativa* Fagaceae (23%) |
| Important minor pollen (3-15%) | *Erica* sp. Ericaceae (7%),<br>*Brassica* sp. Brassicaceae (7%),<br>Liliaceae (6%),<br>*Ceratonia siliqua* Fabaceae (5%),<br>*Rubus* sp. Rosaceae (4%),<br>Apiaceae (4%),<br>*Thymus* sp. Lamiaceae (3%) |
| Minor pollen ( <3%) | *Carthamus lanatus* Asteraceae (2%) |
| Pollen from nectarless plants/Isolated pollen | *Acer* sp. Aceraceae,<br>*Phlomis fruticosa* Lamiaceae,<br>*Olea europaea* Oleaceae,<br>*Cistus* sp. Cistaceae<br>*Hypericum* sp. Hypericaceae,<br>*Helianthemum* sp. Cistaceae, |

**Table S9.** Sample no. 10: Flower/Thyme honey from Hania (Crete).

| | |
|---|---|
| Predominant pollen (>45%) | |
| Secondary pollen (16-45%) | *Castanea sativa* Fagaceae (42%),<br>*Ballota* sp. Lamiaceae (19%) |
| Important minor pollen (3-15%) | *Thymus* sp. Lamiaceae (10%),<br>*Erica* sp. Ericaceae (10%),<br>Asteraceae (4%),<br>*Brassica* sp. Brassicaceae (3%) |
| Minor pollen ( <3%) | Pyrus/Prunus Rosaceae (2%),<br>Apiaceae (2%),<br>*Robinia pseudacacia* Fabaceaea (2%),<br>*Ceratonia siliqua* Fabaceae (2%),<br>*Vicia* sp. Fabaceae (2%) |
| Pollen from nectarless plants/Isolated pollen | *Acer* sp. Aceraceae,<br>*Olea europaea* Oleaceae,<br>*Cistus* sp. Cistaceae<br>*Hypericum* sp. Hypericaceae,<br>*Helianthemum* sp. Cistaceae,<br>*Convolvulus arvensis* Convolvulaceae, |

**Table S10.** Sample no. 11: Asfaka honey from Itea/Galaxidi.

| | |
|---|---|
| Predominant pollen (>45%) | |
| Secondary pollen (16-45%) | *Phlomis Fruticosa* Lamiaceae (38%),<br>*Brassica* sp. Brassicaceae (25%),<br>*Paliurus spina-christi* Rhamnaceae (22%) |
| Important minor pollen (3-15%) | Asteraceae (4%) |
| Minor pollen ( <3%) | Pyrus/Prunus Rosaceae (2%),<br>*Erica* sp. Ericaceae (2%),<br>Apiaceae (2%),<br>*Vicia* sp. Fabaceae (2%) |
| Pollen from nectarless plants/Isolated pollen | Boraginaceae,<br>*Ononis* sp. Fabaceae,<br>*Olea europaea* Oleaceae,<br>*Hypericum* sp. Hypericaceae,<br>*Cistus* sp. Cistaceae,<br>*Ashodelus* sp. Liliaceae,<br>Poaceae, |

**Table S11.** Sample no. 13: Flower honey from Karditsa.

| | |
|---|---|
| Predominant pollen (>45%) | |
| Secondary pollen (16-45%) | *Polygonum aviculare* Polygonaceae (20%) |
| Important minor pollen (3-15%) | *Castanea sativa* Fagaceae (12%),<br>*Paliurus spina-christi* Rhamnaceae (11%),<br>*Trifolium* sp. Fabaceae (10%),<br>Lamiaceae *Thymus* type (9%),<br>*Brassica* sp. Brassicaceae (9%),<br>*Smilax* sp. *Smilacaceae* (8%),<br>*Erica* sp. Ericaceae (7%),<br>Asteraceae (4%),<br>*Hedera helix* Araliaceae (3%)<br>Liliaceae (3%) |
| Minor pollen ( <3%) | *Robinia pseudacacia* Fabaceaea (2%) |
| Pollen from nectarless plants/Isolated pollen | *Cistus* sp. Cistaceae<br>Boraginaceae,<br>*Hypericum* sp. Hypericaceae,<br>Poaceae,<br>Apiaceae,<br>Rosaceae,<br>Gossypium sp. Malvaceae,<br>*Tribulus* sp. Zygophyllaceae |

**Table S12.** Sample no. 16: Flower honey from Heraklion (Crete).

| | |
|---|---|
| Predominant pollen (>45%) | |
| Secondary pollen (16-45%) | *Brassica* sp. Brassicaceae (29%),<br>*Trifolium* sp. Fabaceae (27%),<br>*Ashodelus* sp. Liliaceae (16%), |
| Important minor pollen (3-15%) | *Thymus* sp. Lamiaceae (5%),<br>Apiaceae (4%),<br>*Eucalyptus* sp. Myrtaceae (4%),<br>Pyrus/Prunus Rosaceae (4%),<br>Asteraceae (4%),<br>*Phlomis fruticosa* Lamiaceae (4%) |
| Minor pollen ( <3%) | |
| Pollen from nectarless plants/Isolated pollen | *Acer* sp. Aceraceae,<br>*Olea europaea* Oleaceae,<br>*Cistus* sp. Cistaceae,<br>*Hypericum* sp. Hypericaceae,<br>*Vicia* sp. Fabaceae,<br>*Rubus* sp. Rosaceae,<br>*Convolvulus arvensis* Convolvulaceae |

**Table S13.** Sample no. 22: Flower honey from Thessaly.

| | |
|---|---|
| Predominant pollen (>45%) | *Castanea sativa* Fagaceae (54%) |
| Secondary pollen (16-45%) | |
| Important minor pollen (3-15%) | *Smilax* sp. *Smilacaceae* (12%),<br>*Trifolium* sp. Fabaceae (12%),<br>*Brassica* sp. Brassicaceae (8%),<br>Liliaceae (4%),<br>*Erica* sp. Ericaceae (4%),<br>Pyrus/Prunus Rosaceae (4%) |
| Minor pollen ( <3%) | |
| Pollen from nectarless plants/Isolated pollen | Asteraceae,<br>Apiaceae,<br>Poaceae,<br>*Cistus* sp. Cistaceae,<br>*Ephedra* sp. Ephedraceae,<br>Chenopodiaceae |

**Table S14.** Sample no. 27: Flower honey from Halkidiki.

| | |
|---|---|
| Predominant pollen (>45%) | *Castanea sativa* Fagaceae (78%), |
| Secondary pollen (16-45%) | |
| Important minor pollen (3-15%) | *Helianthus annuus* Asteraceae (6%),<br>*Erica* sp. Ericaceae (4%),<br>*Paliurus spina-christi* Rhamnaceae (4%) |
| Minor pollen ( <3%) | *Trifolium* sp. Fabaceae (2%),<br>Asteraceae (2%),<br>*Brassica* sp. Brassicaceae (2%) |
| Pollen from nectarless plants/Isolated pollen | *Cistus* sp. Cistaceae,<br>*Hypericum* sp. Hypericaceae,<br>Poaceae,<br>Pyrus/Prunus Rosaceae |

**Table S15.** Sample no. 32. Flower honey from Hania (Crete).

| | |
|---|---|
| Predominant pollen (>45%) | |
| Secondary pollen (16-45%) | *Erica* sp. Ericaceae (33%),<br>*Ballota* sp. Lamiaceae (28%) |
| Important minor pollen (3-15%) | *Hedera helix* Araliaceae (10%),<br>*Castanea sativa* Fagaceae (10%),<br>*Trifolium* sp. Fabaceae (10%),<br>*Thymus* sp. Lamiaceae (3%) |
| Minor pollen ( <3%) | *Cirsium* sp. Asteraceae (2%),<br>*Rubus* sp. Rosaceae (2%) |
| Pollen from nectarless plants/Isolated pollen | *Acer* sp. Aceraceae,<br>*Olea europaea* Oleaceae,<br>*Cistus* sp. Cistaceae,<br>*Hypericum* sp. Hypericaceae,<br>Apiaceae,<br>*Ephedra* sp. Ephedraceae,<br>*Verbascum* sp. Scrophulariaceae |

**Table S16.** Sample no. 33: Flower honey from Itea/Galaxidi.

| | |
|---|---|
| Predominant pollen (>45%) | *Castanea sativa* Fagaceae (53%) |
| Secondary pollen (16-45%) | |
| Important minor pollen (3-15%) | *Polygonum aviculare* Polygonaceae (8%),<br>*Hedera helix* Araliaceae (8%),<br>*Brassica* sp. Brassicaceae (7%),<br>Boraginaceae (5%),<br>*Erica* sp. Ericaceae (4%),<br>*Trifolium* sp. Fabaceae (4%),<br>*Ashodelus* sp. Liliaceae (3%),<br>Pyrus/Prunus Rosaceae (3%),<br>Asteraceae (3%) |
| Minor pollen ( <3%) | |
| Pollen from nectarless plants/Isolated pollen | Apiaceae,<br>*Olea europaea* Oleaceae,<br>*Cistus* sp. Cistaceae,<br>*Quercus ilex* Fagaceae, |

- ✓ **<u>Fir honey</u>**

**Table S17.** Sample no. 8: Fir honey from Kitheronas (Parnitha, Attica).

| | |
|---|---|
| Predominant pollen (>45%) | |
| Secondary pollen (16-45%) | *Trifolium* sp. Fabaceae (38%) |
| Important minor pollen (3-15%) | *Polygonum aviculare* Polygonaceae (15%),<br>*Brassica* sp. Brassicaceae (10%),<br>Liliaceae (9%),<br>*Erica* sp. Ericaceae (8%),<br>Asteraceae (7%),<br>*Hedera helix* Araliaceae (6%),<br>Apiaceae (4%) |
| Minor pollen ( <3%) | |
| Pollen from nectarless plants/Isolated pollen | *Olea europaea* Oleaceae,<br>*Cistus* sp. Cistaceae,<br>*Quercus ilex* Fagaceae,<br>*Helianthemum* sp. Cistaceae,<br>Lamiaceae (*Thymus* type),<br>Myrtaceae,<br>*Convolvulus arvensis* Convolvulaceae,<br>*Centaurea* sp. Asteraceae,<br>Poaceae,<br>*Ephedra* sp. Ephedraceae, |

**Table S18.** Sample no. 21: Fir honey from Parnonas (Arkadia).

| | |
|---|---|
| Predominant pollen (>45%) | |
| Secondary pollen (16-45%) | *Trifolium* sp. Fabaceae (31%),<br>Boraginaceae (31%) |
| Important minor pollen (3-15%) | *Castanea sativa* Fagaceae (14%),<br>*Brassica* sp. Brassicaceae (6%),<br>*Thymus* type (3%) |
| Minor pollen ( <3%) | *Erica* sp. Ericaceae (2%),<br>Apiaceae (2%),<br>Asteraceae (2%),<br>Pyrus/Prunus Rosaceae (2%),<br>*Polygonum aviculare* Polygonaceae (2%),<br>*Ononis* sp. Fabaceae (2%) |
| Pollen from nectarless plants/Isolated pollen | *Paliurus spina-christi* Rhamnaceae,<br>*Rubus* sp. Rosaceae,<br>*Ashodelus* sp. Liliaceae,<br>*Cistus* sp. Cistaceae,<br>*Ephedra* sp. Ephedraceae,<br>*Convolvulus arvensis* Convolvulaceae<br>Poaceae,<br>*Olea europaea* Oleaceae,<br>*Helianthemum* sp. Cistaceae,<br>*Hypericum* sp. Hypericaceae |

**Table S19.** Sample no. 23: Fir honey from Karpenisi (Aitoloakarnania).

| | |
|---|---|
| Predominant pollen (>45%) | |
| Secondary pollen (16-45%) | *Castanea sativa* Fagaceae (33%),<br>*Erica* sp. Ericaceae (28%),<br>*Brassica* sp. Brassicaceae (16%) |
| Important minor pollen (3-15%) | *Arbutus* sp. Ericaceae (8%),<br>*Vicia* sp. Fabaceae (8%),<br>Liliaceae (4%) |
| Minor pollen ( <3%) | |
| Pollen from nectarless plants/Isolated pollen | Boraginaceae,<br>*Trifolium* sp. Fabaceae ,<br>*Cistus* sp. Cistaceae,<br>Poaceae,<br>*Quercus ilex* Fagaceae,<br>*Rubus* sp. Rosaceae |

**Table S20.** Sample no. 24: Fir honey from Karpenisi (Aitoloakarnania).

| | |
|---|---|
| Predominant pollen (>45%) | *Castanea sativa* Fagaceae (54%), |
| Secondary pollen (16-45%) | |
| Important minor pollen (3-15%) | *Trifolium* sp. Fabaceae (9%),<br>*Brassica* sp. Brassicaceae (7%),<br>*Polygonum aviculare* Polygonaceae (7%),<br>*Paliurus spina-christi* Rhamnaceae (5%),<br>*Rubus* sp. Rosaceae (4%) |
| Minor pollen ( <3%) | *Vicia* sp. Fabaceae (2%),<br>Pyrus/Prunus Rosaceae (2%),<br>*Erica* sp. Ericaceae (2%),<br>*Citrus* sp. Rutaceae (2%),<br>*Hedera helix* Araliaceae (2%) |
| Pollen from nectarless plants/Isolated pollen | *Hypericum* sp. Hypericaceae,<br>*Quercus ilex* Fagaceae,<br>*Cistus* sp. Cistaceae,<br>*Ephedra* sp. Ephedraceae,<br>*Verbascum* sp. Scrophulariaceae |

**Table S21.** Sample no. 47: Fir honey from Karpenisi (Aitoloakarnania).

| | |
|---|---|
| Predominant pollen (>45%) | *Castanea sativa* Fagaceae (55%), |
| Secondary pollen (16-45%) | |
| Important minor pollen (3-15%) | *Trifolium* sp. Fabaceae (10%),<br>*Erica* sp. Ericaceae (10%),<br>*Brassica* sp. Brassicaceae (7%),<br>*Phlomis Fruticosa* Lamiaceae (7%),<br>*Polygonum aviculare* Polygonaceae (5%),<br>Asteraceae (3%) |
| Minor pollen ( <3%) | |
| Pollen from nectarless plants/Isolated pollen | Pyrus/Prunus Rosaceae,<br>*Vicia* sp. Fabaceae,<br>*Quercus ilex* Fagaceae,<br>*Cistus* sp. Cistaceae,<br>*Ephedra* sp. Ephedraceae,<br>Apiaceae |

**Table S22.** Sample no. 51: Fir honey from Vitina (Arkadia).

| | |
|---|---|
| Predominant pollen (>45%) | *Brassica* sp. Brassicaceae (52%) |
| Secondary pollen (16-45%) | *Trifolium* sp. Fabaceae (16%) |
| Important minor pollen (3-15%) | Apiaceae (7%),<br>Pyrus/Prunus Rosaceae (6%),<br>Liliaceae (6%),<br>*Thymus* type (5%),<br>*Erica* sp. Ericaceae (4%) |
| Minor pollen ( <3%) | *Rubus* sp. Rosaceae (2%) |
| Pollen from nectarless plants/Isolated pollen | *Cistus* sp. Cistaceae,<br>*Hypericum* sp. Hypericaceae,<br>*Quercus ilex* Fagaceae,<br>*Olea europaea* Oleaceae, |

**Table S23.** Sample no. 52: Fir honey from Karpenisi (Aitoloakarnania).

| | |
|---|---|
| Predominant pollen (>45%) | *Castanea sativa* Fagaceae (77%) |
| Secondary pollen (16-45%) | |
| Important minor pollen (3-15%) | *Paliurus spina-christi* Rhamnaceae (7%),<br>*Brassica* sp. Brassicaceae (5%),<br>*Trifolium* sp. Fabaceae (4%) |
| Minor pollen ( <3%) | Asteraceae (2%),<br>*Hedera helix* Araliaceae (2%) |
| Pollen from nectarless plants/Isolated pollen | *Cistus* sp. Cistaceae,<br>*Erica* sp. Ericaceae |

**Table S24.** Sample no. 57: Fir honey from Karpenisi.

| | |
|---|---|
| Predominant pollen (>45%) | |
| Secondary pollen (16-45%) | *Castanea sativa* Fagaceae (38%),<br>*Phlomis Fruticosa* Lamiaceae (19%), |
| Important minor pollen (3-15%) | *Brassica* sp. Brassicaceae (12%),<br>*Trifolium* sp. Fabaceae (12%),<br>*Erica* sp. Ericaceae (6%),<br>Apiaceae (6%) |
| Minor pollen ( <3%) | *Thymus* type (2%),<br>Rutaceae (2%) |
| Pollen from nectarless plants/Isolated pollen | *Quercus ilex* Fagaceae, |

| | |
|---|---|
| | *Olea europaea* Oleaceae,<br>*Ephedra* sp. Ephedraceae,<br>Poaceae |

**Table S25.** Sample no. 60: Fir honey from Parnonas (Arkadia).

| | |
|---|---|
| Predominant pollen (>45%) | |
| Secondary pollen (16-45%) | *Polygonum aviculare* Polygonaceae (33%),<br>*Castanea sativa* Fagaceae (18%) |
| Important minor pollen (3-15%) | *Trifolium* sp. Fabaceae (13%),<br>Boraginaceae (13%),<br>*Smilax* sp. Smilacaceae (13%),<br>Apiaceae (5%),<br>*Centaurea* sp. Asteraceae (4%) |
| Minor pollen ( <3%) | |
| Pollen from nectarless plants/Isolated pollen | *Brassica* sp. Brassicaceae,<br>*Quercus ilex* Fagaceae,<br>*Cistus* sp. Cistaceae,<br>Poaceae,<br>*Hypericum* sp. Hypericaceae |

**Table S26.** Sample no. 63: Fir honey from Parnonas (Arkadia).

| | |
|---|---|
| Predominant pollen (>45%) | |
| Secondary pollen (16-45%) | *Castanea sativa* Fagaceae (29%),<br>*Trifolium* sp. Fabaceae (19%),<br>Asteraceae (19%) |
| Important minor pollen (3-15%) | *Robinia pseudoacacia* Fabaceae (12%),<br>Liliaceae (7%),<br>*Erica* sp. Ericaceae (6%),<br>Apiaceae (6%) |
| Minor pollen ( <3%) | |
| Pollen from nectarless plants/Isolated pollen | *Smilax* sp. Smilacaceae,<br>*Cistus* sp. Cistaceae,<br>*Olea europaea* Oleaceae,<br>*Helianthemum* sp. Cistaceae |

**Table S27.** Sample no. 64: Fir honey from Parnonas (Arkadia).

| | |
|---|---|
| Predominant pollen (>45%) | |
| Secondary pollen (16-45%) | *Trifolium* sp. Fabaceae (45%),<br>Boraginaceae (20%) |
| Important minor pollen (3-15%) | *Vicia* sp. Fabaceae (7%),<br>Pyrus/Prunus Rosaceae (5%),<br>*Brassica* sp. Brassicaceae (4%),<br>*Rubus* sp. Rosaceae (4%),<br>*Lonicera* sp. Caprifoliaceaea (4%), |

| | |
|---|---|
| | Apiaceae (3%),<br>*Erica* sp. Ericaceae (3%),<br>Liliaceae (3%) |
| Minor pollen ( <3%) | |
| Pollen from nectarless plants/Isolated pollen | *Cistus* sp. Cistaceae,<br>*Quercus ilex* Fagaceae |

**Table S28.** Sample no. 65: Fir honey from Karpenisi (Aitoloakarnania).

| | |
|---|---|
| Predominant pollen (>45%) | |
| Secondary pollen (16-45%) | *Trifolium* sp. Fabaceae (18%),<br>*Polygonum aviculare* Polygonaceae (16%) |
| Important minor pollen (3-15%) | Boraginaceae (14%),<br>*Brassica* sp. Brassicaceae (13%),<br>*Lonicera* sp. Caprifoliaceaea (7%),<br>*Centaurea* sp. Asteraceae (7%),<br>*Vicia* sp. Fabaceae (6%),<br>Liliaceae (6%),<br>Pyrus/Prunus Rosaceae (5%),<br>Apiaceae (3%),<br>*Phlomis Fruticosa* Lamiaceae (3%) |
| Minor pollen ( <3%) | |
| Pollen from nectarless plants/Isolated pollen | *Erica* sp. Ericaceae<br>*Quercus ilex* Fagaceae, |

| | |
|---|---|
| | *Olea europaea* Oleaceae, |

**Table S29.** Sample no. 70: Fir honey from Karpenisi (Aitoloakarnania).

| | |
|---|---|
| Predominant pollen (>45%) | |
| Secondary pollen (16-45%) | *Castanea sativa* Fagaceae (41%),<br>*Phlomis Fruticosa* Lamiaceae (22%) |
| Important minor pollen (3-15%) | *Brassica* sp. Brassicaceae (12%),<br>*Trifolium* sp. Fabaceae (9%),<br>*Erica* sp. Ericaceae (7%),<br>Apiaceae (6%) |
| Minor pollen ( <3%) | |
| Pollen from nectarless plants/Isolated pollen | *Quercus ilex* Fagaceae,<br>*Olea europaea* Oleaceae,<br>*Ephedra* sp. Ephedraceae,<br>Poaceae |

**Table S30.** Sample no. 71: Fir honey from Karditsa.

| | |
|---|---|
| Predominant pollen (>45%) | *Castanea sativa* Fagaceae (52%) |
| Secondary pollen (16-45%) | *Paliurus spina-christi* Rhamnaceae (18%) |
| Important minor pollen (3-15%) | *Brassica* sp. Brassicaceae (12%),<br>*Trifolium* sp. Fabaceae (7%), |

| | |
|---|---|
| | *Phlomis Fruticosa* Lamiaceae (5%),<br>Asteraceae (3%) |
| Minor pollen ( <3%) | |
| Pollen from nectarless plants/Isolated pollen | *Quercus ilex* Fagaceae Oleaceae,<br>*Cistus* sp. Cistaceae,<br>Boraginaceae,<br>Rosaceae,<br>Liliaceae |

- ✓ **Thyme honey**

**Table S31.** Sample no. 18: Thyme honey from Crete.

| | |
|---|---|
| Predominant pollen (>45%) | |
| Secondary pollen (16-45%) | *Thymus* sp. Lamiaceae (38%),<br>*Ballota* sp. Lamiaceae (18%) |
| Important minor pollen (3-15%) | *Brassica* sp. Brassicaceae (12%),<br>Asteraceae (10%),<br>*Erica* sp. Ericaceae (9%),<br>*Centaurea* sp. Asteraceae (3%),<br>*Hedera helix* Araliaceae (3%),<br>Apiaceae (3%) |
| Minor pollen ( <3%) | *Ceratonia siliqua* Fabaceae (2%) |
| Pollen from nectarless plants/Isolated pollen | *Acer* sp. Aceraceae, |

| | |
|---|---|
| | *Olea europaea* Oleaceae,<br>*Cistus* sp. Cistaceae,<br>*Hypericum* sp. Hypericaceae,<br>*Vicia* sp. Fabaceae,<br>*Quercus ilex* Fagaceae,<br>*Verbascum* sp. Scrophulariaceae |

**Table S32.** Sample no. 19: Thyme honey from Hania (Crete).

| | |
|---|---|
| Predominant pollen (>45%) | |
| Secondary pollen (16-45%) | *Thymus* sp. Lamiaceae (39%),<br>*Ballota* sp. Lamiaceae (15%) |
| Important minor pollen (3-15%) | *Brassica* sp. Brassicaceae (12%),<br>*Ceratonia siliqua* Fabaceae (7%),<br>*Eucalyptus* sp. Myrtaceae (6%),<br>*Phlomis Fruticosa* Lamiaceae (6%),<br>*Citrus* sp. Rutaceae (4%),<br>Asteraceae (4%),<br>*Rubus* sp. Rosaceae (3%) |
| Minor pollen ( <3%) | *Trifolium* sp. Fabaceae (2%) |
| Pollen from nectarless plants/Isolated pollen | *Olea europaea* Oleaceae,<br>*Cistus* sp. Cistaceae,<br>*Hypericum* sp. Hypericaceae,<br>*Quercus ilex* Fagaceae |

**Table S33.** Sample no. 20: Thyme honey from Hania (Crete).

| | |
|---|---|
| Predominant pollen (>45%) | |
| Secondary pollen (16-45%) | *Thymus* sp. Lamiaceae (24%) |
| Important minor pollen (3-15%) | *Erica* sp. Ericaceae (13%),<br>*Ballota* sp. Lamiaceae (11%),<br>*Centaurea* sp. Asteraceae (10%),<br>Asteraceae (9%),<br>Liliaceae (9%),<br>*Phlomis Fruticosa* Lamiaceae (8%),<br>*Trifolium* sp. Fabaceae (7%),<br>*Brassica* sp. Brassicaceae (7%) |
| Minor pollen ( <3%) | |
| Pollen from nectarless plants/Isolated pollen | *Acer* sp. Aceraceae,<br>*Olea europaea* Oleaceae,<br>*Cistus* sp. Cistaceae,<br>*Hypericum* sp. Hypericaceae,<br>*Quercus ilex* Fagaceae,<br>*Verbascum* sp. Scrophulariaceae |

**Table S34.** Sample no. 28: Thyme honey from Hania (Crete).

| | |
|---|---|
| Predominant pollen (>45%) | |
| Secondary pollen (16-45%) | *Thymus* sp. Lamiaceae (39%),<br>*Brassica* sp. Brassicaceae (15%) |
| Important minor pollen (3-15%) | *Ballota* sp. Lamiaceae (14%),<br>*Ceratonia siliqua* Fabaceae (11%),<br>*Centaurea* sp. Asteraceae (5%),<br>Apiaceae (5%),<br>*Trifolium* sp. Fabaceae (3%),<br>*Phlomis Fruticosa* Lamiaceae (3%),<br>*Cirsium* sp. Asteraceae (3%) |
| Minor pollen ( <3%) | |
| Pollen from nectarless plants/Isolated pollen | *Olea europaea* Oleaceae,<br>*Cistus* sp. Cistaceae,<br>*Hypericum* sp. Hypericaceae,<br>*Quercus ilex* Fagaceae,<br>*Verbascum* sp. Scrophulariaceae,<br>*Ephedra* sp. Ephedraceae |

**Table S35.** Sample no. 29: Thyme honey from Hania (Crete).

| | |
|---|---|
| Predominant pollen (>45%) | |
| Secondary pollen (16-45%) | *Thymus* sp. Lamiaceae (24%),<br>*Ballota* sp. Lamiaceae (18%),<br>*Brassica* sp. Brassicaceae (16%),<br>*Ceratonia siliqua* Fabaceae (16%) |
| Important minor pollen (3-15%) | *Centaurea* sp. Asteraceae (5%),<br>Apiaceae (7%),<br>*Trifolium* sp. Fabaceae (5%),<br>*Phlomis Fruticosa* Lamiaceae (3%) |
| Minor pollen ( <3%) | *Erica* sp. Ericaceae (2%),<br>*Cirsium* sp. Asteraceae (2%) |
| Pollen from nectarless plants/Isolated pollen | *Olea europaea* Oleaceae,<br>*Cistus* sp. Cistaceae,<br>*Hypericum* sp. Hypericaceae,<br>*Verbascum* sp. Scrophulariaceae,<br>*Ephedra* sp. Ephedraceae |

**Table S36.** Sample no. 31: Thyme honey from Hania (Crete).

| | |
|---|---|
| Predominant pollen (>45%) | |
| Secondary pollen (16-45%) | *Thymus* sp. Lamiaceae (40%),<br>*Ballota* sp. Lamiaceae (30%),<br>*Trifolium* sp. Fabaceae (16%) |
| Important minor pollen (3-15%) | *Erica* sp. Ericaceae (10%) |
| Minor pollen ( <3%) | |
| Pollen from nectarless plants/Isolated pollen | *Acer* sp. Aceraceae,<br>Asteraceae,<br>*Hedera helix* Araliaceae,<br>Rosaceae,<br>*Olea europaea* Oleaceae,<br>*Cistus* sp. Cistaceae,<br>*Hypericum* sp. Hypericaceae,<br>*Verbascum* sp. Scrophulariaceae |

**Table S37.** Sample no. 43: Thyme honey from Hania (Crete).

| | |
|---|---|
| Predominant pollen (>45%) | |
| Secondary pollen (16-45%) | *Thymus* sp. Lamiaceae (45%),<br>*Erica* sp. Ericaceae (16%) |
| Important minor pollen (3-15%) | *Phlomis Fruticosa* Lamiaceae (11%),<br>*Ballota* sp. Lamiaceae (10%), |

| | |
|---|---|
| | *Trifolium* sp. Fabaceae (7%),<br>*Castanea sativa* Fagaceae (5%),<br>Asteraceae (3%) |
| Minor pollen ( <3%) | Apiaceae (1%) |
| Pollen from nectarless plants/Isolated pollen | *Acer* sp. Aceraceae,<br>*Hedera helix* Araliaceae,<br>*Olea europaea* Oleaceae,<br>*Cistus* sp. Cistaceae,<br>*Hypericum* sp. Hypericaceae,<br>*Quercus ilex* Fagaceae, |

**Table S38.** Sample no. 45: Thyme honey from Hania (Crete).

| | |
|---|---|
| Predominant pollen (>45%) | *Thymus* sp. Lamiaceae (84%) |
| Secondary pollen (16-45%) | |
| Important minor pollen (3-15%) | *Trifolium* sp. Fabaceae (8%) |
| Minor pollen ( <3%) | *Ballota* sp. Lamiaceae (2%),<br>Asteraceae (2%),<br>Apiaceae (2%),<br>*Erica* sp. Ericaceae (1%) |
| Pollen from nectarless plants/Isolated pollen | *Olea europaea* Oleaceae, |

| | |
|---|---|
| | *Hypericum* sp. Hypericaceae,<br>*Verbascum* sp. Scrophulariaceae |

**Table S39.** Sample no. 46. Thyme honey from Hania (Crete).

| | |
|---|---|
| Predominant pollen (>45%) | |
| Secondary pollen (16-45%) | *Thymus* sp. Lamiaceae (32%),<br>*Ballota* sp. Lamiaceae (22%) |
| Important minor pollen (3-15%) | *Trifolium* sp. Fabaceae (8%),<br>*Centaurea* sp. Asteraceae (8%),<br>Apiaceae (8%),<br>*Phlomis Fruticosa* Lamiaceae (5%),<br>*Eucalyptus* sp. Myrtaceae (5%),<br>*Acer* sp. Aceraceae (5%) |
| Minor pollen ( <3%) | *Erica* sp. Ericaceae (2%),<br>*Brassica* sp. Brassicaceae (2%) |
| Pollen from nectarless plants/Isolated pollen | *Olea europaea* Oleaceae,<br>*Hypericum* sp. Hypericaceae, |

**Table S40.** Sample no. 52: Thyme honey from Hania (Crete).

| | |
|---|---|
| Predominant pollen (>45%) | *Thymus* sp. Lamiaceae (53%) |
| Secondary pollen (16-45%) | *Ballota* sp. Lamiaceae (25%) |
| Important minor pollen (3-15%) | *Trifolium* sp. Fabaceae (9%),<br>Asteraceae (5%),<br>*Centaurea* sp. Asteraceae (3%),<br>*Castanea sativa* Fagaceae (3%) |
| Minor pollen ( <3%) | |
| Pollen from nectarless plants/Isolated pollen | *Acer* sp. Aceraceae,<br>*Erica* sp. Ericaceae,<br>*Olea europaea* Oleaceae,<br>*Hypericum* sp. Hypericaceae |

**Table S41.** Sample no. 53: Thyme honey from Hania (Crete).

| | |
|---|---|
| Predominant pollen (>45%) | *Thymus* sp. Lamiaceae (72%) |
| Secondary pollen (16-45%) | |
| Important minor pollen (3-15%) | Asteraceae (7%),<br>*Phlomis Fruticosa* Lamiaceae (6%),<br>Apiaceae (5%),<br>*Ballota* sp. Lamiaceae (3%),<br>*Centaurea* sp. Asteraceae (3%),<br>*Brassica* sp. Brassicaceae (2%) |

| | |
|---|---|
| Minor pollen ( <3%) | |
| Pollen from nectarless plants/Isolated pollen | *Acer* sp. Aceraceae,<br>*Olea europaea* Oleaceae,<br>*Cistus* sp. Cistaceae,<br>*Hypericum* sp. Hypericaceae,<br>*Quercus ilex* Fagaceae |

**Table S42.** Sample no. 58: Thyme honey from Kos Island.

| | |
|---|---|
| Predominant pollen (>45%) | |
| Secondary pollen (16-45%) | Boraginaceae (42%)<br>Liliaceae (22%)<br>*Thymus* sp. Lamiaceae (19%) |
| Important minor pollen (3-15%) | *Eucalyptus* sp. Myrtaceae (8%)<br>*Trifolium* sp. Fabaceae (3%) |
| Minor pollen ( <3%) | Asteraceae (2%),<br>*Brassica* sp. Brassicaceae (2%), |
| Pollen from nectarless plants/Isolated pollen | *Olea europaea* Oleaceae,<br>*Cistus* sp. Cistaceae<br>*Hypericum* sp. Hypericaceae, |

**Table S43.** Sample no. 59: Thyme sample from Kos Island.

| | |
|---|---|
| Predominant pollen (>45%) | |
| Secondary pollen (16-45%) | *Thymus* sp. Lamiaceae (31%),<br>Boraginaceae (28%),<br>Liliaceae (26%) |
| Important minor pollen (3-15%) | *Eucalyptus* sp. Myrtaceae (5%) |
| Minor pollen ( <3%) | Asteraceae (2%),<br>*Brassica* sp. Brassicaceae (2%),<br>*Erica* sp. Ericaceae (2%),<br>*Carthamus lanatus* Asteraceae (2%) |
| Pollen from nectarless plants/Isolated pollen | *Trifolium* sp. Fabaceae ,<br>*Olea europaea* Oleaceae,<br>*Cistus* sp. Cistaceae,<br>*Hypericum* sp. Hypericaceae,<br>Rosaceae |

**Table S44.** Sample no. 62: Thyme honey from Hania (Crete).

| | |
|---|---|
| Predominant pollen (>45%) | |
| Secondary pollen (16-45%) | *Trifolium* sp. Fabaceae (27%),<br>*Thymus* sp. Lamiaceae (21%) |
| Important minor pollen (3-15%) | Asteraceae (12%),<br>*Brassica* sp. Brassicaceae (11%),<br>Rosaceae (10%), |

| | |
|---|---|
| | *Myrtus* sp. Myrtaceae (9%),<br>*Erica* sp. Ericaceae (5%),<br>*Oxalis* sp. Oxalidaceae (3%) |
| Minor pollen ( <3%) | |
| Pollen from nectarless plants/Isolated pollen | *Olea europaea* Oleaceae,<br>*Cistus* sp. Cistaceae,<br>*Hypericum* sp. Hypericaceae |

**Table S45.** Sample no. 67: Thyme honey from Hania (Crete).

| | |
|---|---|
| Predominant pollen (>45%) | |
| Secondary pollen (16-45%) | *Ballota* sp. Lamiaceae (28%)<br>*Thymus* sp. Lamiaceae 25%) |
| Important minor pollen (3-15%) | *Brassica* sp. Brassicaceae (13%),<br>*Ceratonia siliqua* Fabaceae (11%)<br>*Centaurea* sp. Asteraceae (10%),<br>*Erica* sp. Ericaceae (4%)<br>Asteraceae (4%),<br>*Hedera helix* Araliaceae (3%) |
| Minor pollen ( <3%) | |
| Pollen from nectarless plants/Isolated pollen | *Olea europaea* Oleaceae,<br>*Cistus* sp. Cistaceae<br>*Hypericum* sp. Hypericaceae,<br>*Verbascum* sp. Scrophulariaceae |

| | |
|---|---|
| | |

- ✓ **Pine honey**

**Table S46.** Sample no. 25: Pine honey from Halkidiki.

| | |
|---|---|
| Predominant pollen (>45%) | *Castanea sativa* Fagaceae (68%) |
| Secondary pollen (16-45%) | |
| Important minor pollen (3-15%) | *Smilax* sp. *Smilacaceae* (9%),<br>*Erica* sp. Ericaceae (9%),<br>*Trifolium* sp. Fabaceae (7%),<br>*Polygonum aviculare* Polygonaceae (3%) |
| Minor pollen ( <3%) | Liliaceae (2%) |
| Pollen from nectarless plants/Isolated pollen | *Cistus* sp. Cistaceae,<br>Chenopodiaceae,<br>*Hypericum* sp. Hypericaceae |
| HDE/P | 0,25 |

**Table S47.** Sample no. 34: Pine honey from Thasos.

| | |
|---|---|
| Predominant pollen (>45%) | *Castanea sativa* Fagaceae (50%) |
| Secondary pollen (16-45%) | *Paliurus spina-christi* Rhamnaceae (35%) |
| Important minor pollen (3-15%) | *Erica* sp. Ericaceae (7%) |
| Minor pollen ( <3%) | *Trifolium* sp. Fabaceae (2%),<br>Liliaceae (2%),<br>*Polygonum aviculare* Polygonaceae (1%),<br>*Helianthus annuus* Asteraceae (1%) |
| Pollen from nectarless plants/Isolated pollen | Pyrus/Prunus Rosaceae<br>*Cistus* sp. Cistaceae,<br>*Brassica* sp. Brassicaceae<br>Asteraceae<br>*Vicia* sp. Fabaceae<br>*Olea europaea* Oleaceae,<br>Poaceae,<br>*Ephedra* sp. Ephedraceae, |
| HDE/P | 0,25 |

**Table S48**. Sample no. 35: Pine honey from Thasos.

| | |
|---|---|
| Predominant pollen (>45%) | *Castanea sativa* Fagaceae (59%) |
| Secondary pollen (16-45%) | *Paliurus spina-christi* Rhamnaceae (28%) |
| Important minor pollen (3-15%) | |
| Minor pollen ( <3%) | *Erica* sp. Ericaceae (3%),<br>Apiaceae (2%),<br>*Helianthus annuus* Asteraceae (1%),<br>*Vicia* sp. Fabaceae (1%),<br>*Trifolium* sp. Fabaceae (1%),<br>*Thymus* sp. Lamiaceae (1%),<br>*Rubus* sp. Rosaceae (1%),<br>*Centaurea* sp. Asteraceae (1%) |
| Pollen from nectarless plants/Isolated pollen | Pyrus/Prunus Rosaceae<br>*Cistus* sp. Cistaceae,<br>Asteraceae,<br>Poaceae |
| HDE/P | 0,30 |

**Table S49.** Sample no. 36: Pine honey from Thasos.

| | |
|---|---|
| Predominant pollen (>45%) | *Castanea sativa* Fagaceae (52%) |
| Secondary pollen (16-45%) | *Paliurus spina-christi* Rhamnaceae (20%) |
| Important minor pollen (3-15%) | *Helianthus annuus* Asteraceae (9%),<br>Boraginaceae (4%),<br>*Erica* sp. Ericaceae (4%),<br>Asteraceae (4%) |
| Minor pollen ( <3%) | *Brassica* sp. Brassicaceae (2%),<br>Pyrus/Prunus Rosaceae (2%),<br>*Trifolium* sp. Fabaceae (2%) |
| Pollen from nectarless plants/Isolated pollen | *Cistus* sp. Cistaceae,<br>Poaceae,<br>Chenopodiaceae |
| HDE/P | 0,25 |

**Table S50.** Sample no. 37: Pine honey from Thasos.

| | |
|---|---|
| Predominant pollen (>45%) | |
| Secondary pollen (16-45%) | *Paliurus spina-christi* Rhamnaceae (43%)<br>*Castanea sativa* Fagaceae (34%) |
| Important minor pollen (3-15%) | Liliaceae (7%) |
| Minor pollen ( <3%) | *Helianthus annuus* Asteraceae (2%),<br>*Erica* sp. Ericaceae (2%),<br>Pyrus/Prunus Rosaceae (2%),<br>Apiaceae (2%),<br>*Trifolium* sp. Fabaceae (2%),<br>*Brassica* sp. Brassicaceae (2%),<br>*Rubus* sp. Rosaceae (2%) |
| Pollen from nectarless plants/Isolated pollen | *Cistus* sp. Cistaceae,<br>Poaceae,<br>*Ephedra* sp. Ephedraceae,<br>*Olea europaea* Oleaceae,<br>*Hypericum* sp. Hypericaceae,<br>*Verbascum* sp. Scrophulariaceae,<br>*Quercus ilex* Fagaceae,<br>*Pinus* sp. Pinaceae |
| HDE/P | 0,25 |

**Table S51.** Sample no. 39: Pine honey from Thasos.

| | |
|---|---|
| Predominant pollen (>45%) | |
| Secondary pollen (16-45%) | *Castanea sativa* Fagaceae (81%) |
| Important minor pollen (3-15%) | *Erica* sp. Ericaceae (7%),<br>Liliaceae (4%),<br>Asteraceae (3%),<br>,*Paliurus spina-christi* Rhamnaceae (3%) |
| Minor pollen ( <3%) | |
| Pollen from nectarless plants/Isolated pollen | Pyrus/Prunus Rosaceae,<br>*Cistus* sp. Cistaceae,<br>Poaceae,<br>*Hypericum* sp. Hypericaceae |
| HDE/P | 0,19 |

**Table S52.** Sample no. 40: Pine honey from Thasos.

| | |
|---|---|
| Predominant pollen (>45%) | *Castanea sativa* Fagaceae (64%) |
| Secondary pollen (16-45%) | *Paliurus spina-christi* Rhamnaceae (21%) |
| Important minor pollen (3-15%) | *Erica* sp. Ericaceae (5%),<br>Asteraceae (3%),<br>*Centaurea* sp. Asteraceae (3%) |

| | |
|---|---|
| Minor pollen ( <3%) | *Helianthus annuus* Asteraceae (2%) |
| Pollen from nectarless plants/Isolated pollen | *Trifolium* sp. Fabaceae,<br>*Brassica* sp. Brassicaceae,<br>*Cistus* sp. Cistaceae,<br>*Ephedra* sp. Ephedraceae,<br>*Olea europaea* Oleaceae,<br>*Quercus ilex* Fagaceae,<br>Chenopodiaceae |
| HDE/P | 0,19 |

**Table S53.** Sample no. 41: Pine honey from Thasos.

| | |
|---|---|
| Predominant pollen (>45%) | *Castanea sativa* Fagaceae (63%) |
| Secondary pollen (16-45%) | |
| Important minor pollen (3-15%) | *Helianthus annuus* Asteraceae (7%),<br>Boraginaceae (5%),<br>*Paliurus spina-christi* Rhamnaceae (6%),<br>Liliaceae (5%),<br>*Trifolium* sp. Fabaceae (5%),<br>Apiaceae (5%) |
| Minor pollen ( <3%) | *Erica* sp. Ericaceae (2%) |

| | |
|---|---|
| Pollen from nectarless plants/Isolated pollen | *Quercus ilex* Fagaceae,<br>*Cistus* sp. Cistaceae,<br>*Vicia* sp. Fabaceae,<br>*Brassica* sp. Brassicaceae |
| HDE/P | 0,20 |

**Table S54.** Sample no. 42: Pine honey from Thasos.

| | |
|---|---|
| Predominant pollen (>45%) | |
| Secondary pollen (16-45%) | *Erica* sp. Ericaceae (42%) |
| Important minor pollen (3-15%) | *Paliurus spina-christi* Rhamnaceae (15%)<br>*Castanea sativa* Fagaceae (10%)<br>*Helianthus annuus* Asteraceae (10%),<br>*Trifolium* sp. Fabaceae (5%)<br>Pyrus/Prunus Rosaceae (5%)<br>Liliaceae (5%)<br>Apiaceae (5%), |
| Minor pollen ( <3%) | |
| Pollen from nectarless plants/Isolated pollen | *Quercus ilex* Fagaceae,<br>*Cistus* sp. Cistaceae,<br>Poaceae,<br>*Pinus* sp. Pinaceae |
| HDE/P | 0,21 |

**Table S55.** Sample no. 48: Pine honey from Halkidiki.

| | |
|---|---|
| Predominant pollen (>45%) | *Castanea sativa* Fagaceae (80%) |
| Secondary pollen (16-45%) | |
| Important minor pollen (3-15%) | *Polygonum aviculare* Polygonaceae (8%),<br>*Erica* sp. Ericaceae (4%),<br>*Trifolium* sp. Fabaceae (4%),<br>*Brassica* sp. Brassicaceae (2%) |
| Minor pollen ( <3%) | *Ashodelus* sp. Liliaceae (1%) |
| Pollen from nectarless plants/Isolated pollen | Asteraceae,<br>*Smilax* sp. *Smilacaceae,*<br>*Hypericum* sp. Hypericaceae,<br>*Quercus ilex* Fagaceae,<br>*Arbutus* sp. Ericaceae,<br>Poaceae,<br>*Ephedra* sp. Ephedraceae |
| HDE/P | 1.02 |

**Table S56.** Sample no. 49: Pine honey from Halkidiki.

| | |
|---|---|
| Predominant pollen (>45%) | |
| Secondary pollen (16-45%) | *Castanea sativa* Fagaceae (35%),<br>*Paliurus spina-christi* Rhamnaceae (28%),<br>*Helianthus annuus* Asteraceae (16%) |
| Important minor pollen (3-15%) | *Trifolium* sp. Fabaceae (5%),<br>*Brassica* sp. Brassicaceae (4%) |
| Minor pollen ( <3%) | *Erica* sp. Ericaceae (2%),<br>Apiaceae (2%),<br>Liliaceae (2%)<br>Asteraceae (2%)<br>*Polygonum aviculare* Polygonaceae (1%),<br>*Hedera helix* Araliaceae (1%) |
| Pollen from nectarless plants/Isolated pollen | Poaceae,<br>*Hypericum* sp. Hypericaceae,<br>*Convolvulus arvensis* Convolvulaceae,<br>Chenopodiaceae |
| HDE/P | 0,47 |

**Table S57.** Sample no. 50: Pine honey from Halkidiki.

| | |
|---|---|
| Predominant pollen (>45%) | |
| Secondary pollen (16-45%) | *Castanea sativa* Fagaceae (51%),<br>*Paliurus spina-christi* Rhamnaceae (39%) |
| Important minor pollen (3-15%) | |
| Minor pollen ( <3%) | Liliaceae (2%),<br>Asteraceae (2%),<br>*Polygonum aviculare* Polygonaceae (2%),<br>Apiaceae (1%),<br>*Trifolium* sp. Fabaceae (1%) |
| Pollen from nectarless plants/Isolated pollen | *Convolvulus arvensis* Convolvulaceae, |
| HDE/P | 0,78 |

**Table S58.** Sample no. 55: Pine honey from Evia.

| | |
|---|---|
| Predominant pollen (>45%) | *Brassica* sp. Brassicaceae (46%) |
| Secondary pollen (16-45%) | *Paliurus spina-christi* Rhamnaceae (20%) |
| Important minor pollen (3-15%) | Liliaceae (11%),<br>*Trifolium* sp. Fabaceae (7%),<br>Boraginaceae (6%),<br>Apiaceae (3%), |

| | |
|---|---|
| | Pyrus/Prunus Rosaceae (3%) |
| Minor pollen ( <3%) | *Smilax* sp. *Smilacaceae* (2%) |
| Pollen from nectarless plants/Isolated pollen | Asteraceae,<br>*Convolvulus arvensis* Convolvulaceae,<br>*Hedera helix* Araliaceae,<br>*Erica* sp. Ericaceae |
| HDE/P | 0,57 |

**Table S59.** Sample no. 61: Pine honey from Tripoli (Arkadia).

| | |
|---|---|
| Predominant pollen (>45%) | |
| Secondary pollen (16-45%) | *Polygonum aviculare* Polygonaceae (36%),<br>Boraginaceae (17%),<br>*Trifolium* sp. Fabaceae (15%) |
| Important minor pollen (3-15%) | Asteraceae (6%),<br>Apiaceae (5%),<br>*Centaurea* sp. Asteraceae (4%),<br>*Smilax* sp. *Smilacaceae* (4%),<br>*Brassica* sp. Brassicaceae (4%),<br>*Castanea sativa* Fagaceae (3%),<br>*Thymus* type Lamiaceae (3%) |

| | |
|---|---|
| Minor pollen ( <3%) | |
| Pollen from nectarless plants/Isolated pollen | *Quercus ilex* Fagaceae,<br>*Cistus* sp. Cistaceae,<br>Chenopodiaceae<br>*Hypericum* sp. Hypericaceae, |
| HDE/P | 1.07 |

**HDE/P**: Honeydew elements per pollen grains.

**Tables S60 to S61:** MANOVA analysis results.

**Table S60.** Significance of the ATR-FTIR spectral regions based on their transmittance values, for the botanical origin confirmation of monofloral and polyfloral honey

| Tests of Equality of Group Means | | | | | |
|---|---|---|---|---|---|
| Transmittance values of the spectral regions | Wilks' Lambda | F | df1 | df2 | Sig. |
| Region A (3300-3200) | 0.308 | 41.141 | 3 | 55 | 0.000 |
| Region B (2970-2920) | 0.793 | 4.795 | 3 | 55 | 0.005 |
| Region C (1730-1600) | 0.341 | 35.412 | 3 | 55 | 0.000 |
| Region D (1420-1410) | 0.296 | 43.644 | 3 | 55 | 0.000 |
| Region E (1390-1380) | 0.329 | 37.313 | 3 | 55 | 0.000 |
| Region F1 (1380-1330) | 0.410 | 26.343 | 3 | 55 | 0.000 |
| Region F2 (1380-1330) | 0.260 | 52.157 | 3 | 55 | 0.000 |
| Region G1 (1260-1225) | 0.163 | 94.421 | 3 | 55 | 0.000 |
| Region G2 (1260-1225) | 0.348 | 34.295 | 3 | 55 | 0.000 |
| Region H (1210-1180) | 0.403 | 27.167 | 3 | 55 | 0.000 |
| Region I (1150-1130) | 0.306 | 41.590 | 3 | 55 | 0.000 |
| Region J1 (1100-1010) | 0.638 | 10.418 | 3 | 55 | 0.000 |
| Region J2 (1100-1010) | 0.450 | 22.370 | 3 | 55 | 0.000 |
| Region J3 (1100-1010) | 0.658 | 9.511 | 3 | 55 | 0.000 |
| Region K1 (950-750) | 0.749 | 6.133 | 3 | 55 | 0.001 |
| Region K2 (950-750) | 0.977 | 0.422 | 3 | 55 | 0.738 |
| Region K3 (950-750) | 0.436 | 23.725 | 3 | 55 | 0.000 |
| Region K4 (950-750) | 0.459 | 21.570 | 3 | 55 | 0.000 |

Sig. : Significance (*p*-value).

**Table S61.** Significance of the ATR-FTIR spectral regions for the botanical origin confirmation of monofloral honey adulterated with flower/polyfloral honey

| Tests of Equality of Group Means | | | | | |
|---|---|---|---|---|---|
| Transmittance values of the spectral regions | Wilks' Lambda | F | df1 | df2 | Sig. |
| Region A (3300-3200) | 0.311 | 35.015 | 6 | 95 | 0.000 |
| Region B (2970-2920) | 0.694 | 6.989 | 6 | 95 | 0.000 |
| Region C (1730-1600) | 0.345 | 30.122 | 6 | 95 | 0.000 |
| Region D (1420-1410) | 0.301 | 36.792 | 6 | 95 | 0.000 |
| Region E (1390-1380) | 0.324 | 33.023 | 6 | 95 | 0.000 |
| Region F1 (1380-1330) | 0.416 | 22.267 | 6 | 95 | 0.000 |
| Region F2 (1380-1330) | 0.280 | 40.675 | 6 | 95 | 0.000 |
| Region G1 (1260-1225) | 0.204 | 61.596 | 6 | 95 | 0.000 |

| | | | | | |
|---|---|---|---|---|---|
| Region G2 (1260-1225) | 0.350 | 29.434 | 6 | 95 | 0.000 |
| Region H (1210-1180) | 0.436 | 20.452 | 6 | 95 | 0.000 |
| Region I (1150-1130) | 0.311 | 35.103 | 6 | 95 | 0.000 |
| Region J1 (1100-1010) | 0.527 | 14.193 | 6 | 95 | 0.000 |
| Region J2 (1100-1010) | 0.340 | 30.674 | 6 | 95 | 0.000 |
| Region J3 (1100-1010) | 0.617 | 9.843 | 6 | 95 | 0.000 |
| Region K1 (950-750) | 0.530 | 14.016 | 6 | 95 | 0.000 |
| Region K2 (950-750) | 0.889 | 1.971 | 6 | 95 | 0.077 |
| Region K3 (950-750) | 0.480 | 17.135 | 6 | 95 | 0.000 |
| Region K4 (950-750) | 0.465 | 18.243 | 6 | 95 | 0.000 |

Sig. : Significance (*p*-value).
